# A Combinatorial Regulatory Platform Determines Expression of RNA Polymerase III Subunit RPC7α (*POLR3G*) in Cancer

**DOI:** 10.3390/cancers15204995

**Published:** 2023-10-15

**Authors:** Ruiying Cheng, Sihang Zhou, Rajendra K C, Simon Lizarazo, Leela Mouli, Anshita Jayanth, Qing Liu, Kevin Van Bortle

**Affiliations:** 1 Department of Cell and Developmental Biology, University of Illinois Urbana-Champaign, Urbana, IL 61801, USA; rcheng14@illinois.edu (R.C.); sihangz2@illinois.edu (S.Z.); 2 Center for Biophysics and Quantitative Biology, University of Illinois Urbana-Champaign, Urbana, IL 61801, USA; rkc5@illinois.edu; 3 Department of Molecular and Integrative Physiology, University of Illinois Urbana-Champaign, Urbana, IL 61801, USA; simonl3@illinois.edu; 4 School of Molecular and Cellular Biology, University of Illinois Urbana-Champaign, Urbana, IL 61801, USA; lmouli2@illinois.edu (L.M.); jayanth3@illinois.edu (A.J.); 5 Department of Biological Sciences, Clemson University, Clemson, SC 29634, USA; qliu4@clemson.edu; 6 Center for Human Genetics, Clemson University, Greenwood, SC 29646, USA; 7 Cancer Center at Illinois, University of Illinois Urbana-Champaign, Urbana, IL 61801, USA

**Keywords:** Pol III, RPC7, RPC7α, RPC7β, POLR3G, POLR3GL, RPC32, RPC32α, RPC32β, tRNA

## Abstract

**Simple Summary:**

The RNA polymerase III complex incorporates two forms of subunit RPC7: RPC7α, which is highly abundant during early development, and RPC7β, the constitutive form of RPC7 in most tissues. Here, we investigate the gene regulatory mechanisms that give rise to high RPC7α levels in cancer, which is linked with unfavorable outcomes in patients. Our survey points to a gene-internal regulatory element and identifies a multitude of transcription factors that contribute to RPC7α abundance, altogether establishing a combinatorial model for Pol III identity in cancer.

**Abstract:**

RNA polymerase III (Pol III) subunit RPC7α, which is encoded by *POLR3G* in humans, has been linked to both tumor growth and metastasis. Accordantly, high *POLR3G* expression is a negative prognostic factor in multiple cancer subtypes. To date, the mechanisms underlying *POLR3G* upregulation have remained poorly defined. We performed a large-scale genomic survey of mRNA and chromatin signatures to predict drivers of *POLR3G* expression in cancer. Our survey uncovers positive determinants of *POLR3G* expression, including a gene-internal super-enhancer bound with multiple transcription factors (TFs) that promote *POLR3G* expression, as well as negative determinants that include gene-internal DNA methylation, retinoic-acid induced differentiation, and MXD4-mediated disruption of *POLR3G* expression. We show that novel TFs identified in our survey, including ZNF131 and ZNF207, functionally enhance *POLR3G* expression, whereas MXD4 likely obstructs MYC-driven expression of *POLR3G* and other growth-related genes. Integration of chromatin architecture and gene regulatory signatures identifies additional factors, including histone demethylase KDM5B, as likely influencers of *POLR3G* gene activity. Taken together, our findings support a model in which *POLR3G* expression is determined with multiple factors and dynamic regulatory programs, expanding our understanding of the circuitry underlying *POLR3G* upregulation and downstream consequences in cancer.

## 1. Introduction

The RNA polymerase III (Pol III) transcriptome includes multiple classes of small noncoding RNA (ncRNA) with central roles in transcription regulation, translation, RNA processing, and other core cellular processes. Increased levels of Pol III activity and Pol III-derived ncRNA, which are integral to protein accumulation and cell growth, are commonly observed in cancer and other disease contexts [1]. Pol III transcription is regulated through a variety of mechanisms, including growth signaling events that converge on the expression or post-translational modification of specific transcription factors, dynamic repression by regulatory factor MAF1, or changes in the Pol III machinery itself [2]. However, despite accumulating evidence of dysregulation in cancer, our current understanding of the specific mechanisms driving Pol III overactivity in cancer remains limited.

Pol III structurally incorporates two stably associated subcomplexes that play essential functions in Pol III transcription initiation, elongation, and termination [3]. The ternary RPC3-RPC6-RPC7 subcomplex, for example, interacts with the core transcription factor TFIIIB to recruit the Pol III complex to target genes [4,5]. Within the RPC3-RPC6-RPC7 heterotrimer, subunit RPC7 establishes interactions with the stalk module that are thought to function in conformational changes in Pol III structure during transcription initiation. Disruption or deletion of the highly conserved RPC7 stalk bridge interface motif is lethal in *S. cerevisiae*, suggesting an essential role in Pol III transcription [6]. In humans, the RPC7 subunit is encoded by two paralogous genes, *POLR3G* and *POLR3GL*, evolutionarily derived from a gene duplication event in the common ancestor of vertebrates [7]. The mutually exclusive incorporation of either RPC7α (POLR3G) or RPC7β (POLR3GL) distinguishes two forms of RNA polymerase III, Pol IIIα and Pol IIIβ, first identified in mouse myeloma tumor cells [8] and subsequently discovered in human cells [9]. The human forms of RPC7α and RPC7β share 46% amino acid identity and recent evidence suggests differences in RPC7 subunit incorporation may shape Pol III activity with implications for cellular growth, proliferation, and cancer progression [7,10,11,12,13].

*POLR3G* expression is generally restricted to the earliest stages of development but re-emerges in cancer, in contrast to more constitutive expression patterns observed for *POLR3GL* and genes encoding other Pol III subunits [10]. Notably, high *POLR3G* expression is associated with poor survival outcomes in a variety of cancers, including transitional cell carcinoma [14], multiple myeloma [15], hepatocellular carcinoma [16], and lung adenocarcinoma [17]. Overexpression of *POLR3GL*, on the other hand, is not associated with unfavorable outcomes, suggesting *POLR3G* expression and the re-emergence of RPC7α establishes a unique form of Pol III with implications for disease progression and clinical outcomes [12]. Thus, understanding and potentially targeting *POLR3G* expression may present a promising strategy for disrupting Pol III-driven growth in cancer contexts. 

During early development, *POLR3G* transcription is driven by pluripotency factors OCT4 (POU5F1) and NANOG, which directly occupy regulatory elements proximal to the *POLR3G* transcription start site [18]. However, the oncogenic transcription factor MYC also localizes to the *POLR3G* gene promoter in multiple cell lines and cancer contexts, presumably in the absence of OCT4 and NANOG, suggesting Pol III identity may be shaped by a distinct transcription factor repertoire in disease contexts [7,12]. Nevertheless, NANOG regulates *POLR3G* expression in prostate cancer cells [10], indicating that *POLR3G* mRNA levels may be driven by multiple transcription factors and regulatory programs. Understanding the breadth and potential intersection of transcription factors that regulate *POLR3G* expression is therefore important for deconstructing the mechanisms of *POLR3G* upregulation and downstream consequences in cancer.

Here, we perform a genomic survey of both the transcription and chromatin signatures associated with *POLR3G* mRNA abundance in cancer with the goal of uncovering gene regulatory mechanisms contributing to *POLR3G* expression. We complement our findings with analyses of TF-binding patterns proximal to *POLR3G*, as well as functional experiments targeting multiple transcription factors. Our results point to a multi-layered regulatory structure that juxtaposes broad-acting factors, such as MYC, with a combination of transcription factors and gene-internal enhancer elements linked with *POLR3G* upregulation in cancer.

## 2. Materials and Methods

### 2.1. Cell Lines

THP−1 cells were obtained from ATCC (Batch# 62454382) and propagated in T-75 flasks between 0.2 and 1 × 10^6^ cells/mL in an RPMI-1640 (Catalog# 11875093, Gibco, Billings, MT, USA) growth medium. THP−1 experiments were conducted on cells between passage 10 and 15. HEK293T cells were obtained from ATCC (CRL-3216, Batch# 70049877) and grown in 10 cm BioLite™ Cell Culture Treated Dishes (Catalog# 12-556-002, Thermo Scientific, Waltham, MA, USA) in Dulbecco’s Modified Eagle Medium, high glucose (Catalog# 11965092, Gibco, Billings, MT, USA). HEK293T experiments were conducted on cells between passage 20 and 25. A549 cells were obtained from ATCC (CRM-CCL-185, Batch# 70045215) and grown in 10 cm BioLite™ Cell Culture Treated Dishes in Ham’s F-12K (Kaighn’s) Medium (Catalog# 21127022, Gibco, Billings, MT, USA). A549 experiments were conducted on cells between passage 10 and 15. H1 human embryonic stem cells (H1-hESCs) were obtained from WiCell (Madison, Wisconsin, USA Cat# WAe001-A) and grown in Matrigel (Catalog# 354277, Corning, Corning, New York, USA.)-coated 12-well plates in an Essential 8™ Medium (Catalog# A1517001, Gibco, Billings, MT, USA). Each culture medium was supplemented with 10% fetal bovine serum and 1% penicillin–streptomycin. Cells were kept in a humidified atmosphere at 37 °C with 5% CO_2_. 

### 2.2. Reagents and Chemicals

**Figure 1:**
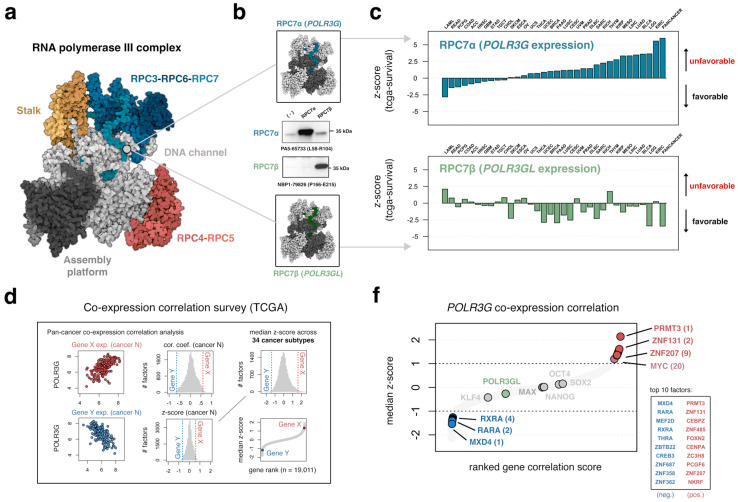

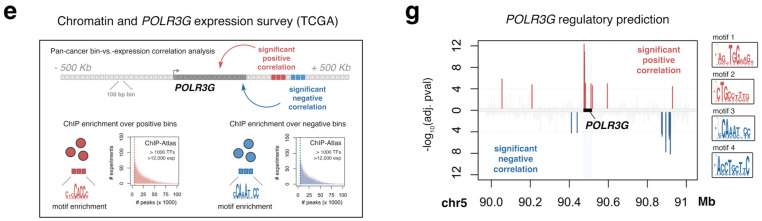
An integrated survey of mRNA and chromatin correlates identifies candidate regulatory factors and sequence elements. (**a**) Cryo-EM structure highlighting major elements of the Pol III complex, including the stalk module (gold), RPC4-5 subcomplex (red), and RPC3-RPC6-RPC7 subcomplex (blue). Structure 7D59 [6] re-colored in Mol*. (**b**) RPC7 subunits (blue/green) are highlighted for generalization; true structure corresponds to subunit RPC7α. Immunoblot analysis of commercial antibody specificity for RPC7α (*POLR3G*) and RPC7β (*POLR3GL*) in HEK293T cell lysates with ectopic *POLR3G* or *POLR3GL* overexpression. (−) non-transfected HEK293T cells. (**c**) Clinical outcome signatures for *POLR3G* and *POLR3GL* expression stratified by cancer subtype (*x*-axis) and sorted by *POLR3G* z-score (*y*-axis) association with favorable or unfavorable outcomes. Data acquired from tcga-survival.com version 2.0 [19]. (**d**) Approach: co-expression correlation survey determines subtype and pan-cancer correlation scores between *POLR3G* and all other genes, with the goal of identifying candidate regulators broadly involved in *POLR3G* activity. (**e**) Approach: chromatin survey identifies 100 bp bins (±500 Kb) with significant correlation scores between chromatin accessibility and *POLR3G* expression in cancer, towards identifying candidate regulatory sequences related to *POLR3G* upregulation. (**f**) Co-expression correlation results from Figure 1d: correlations are ranked (*x*-axis) and z-transformed (*y*-axis). Pluripotency factors (OCT4, NANOG, SOX2, and KLF4) and MAX are highlighted in gray. Significant positive (red) and negative (blue) correlates include ZNF131, ZNF207, and RARA and RXRA. Table highlights top 10 positive and negative correlates after subsetting for chromatin/transcription factors. (**g**) Chromatin correlation results from Figure 1e: the most significant positive (red) DNA elements are identified within *POLR3G*, whereas significant negative (blue) DNA correlates are identified distal-downstream. Bins are scored with −log_10_(adjusted correlation *p*-value); regions with negative correlation coefficients are inverted along the *y*-axis. The top enriched motifs within positive (red) and negative (blue) bins were identified with STREME [32].

10058-F4 (Catalog#HY-12702, MedChemExpress, Monmouth Junction, NJ, USA) was dissolved in DMSO (Catalog# BP231-100, FisherScientific, Waltham, MA, USA) and added to a final concentration of 50 μM, 100 μM, 150 μM, or 200 μM. ATRA (Cat. No.: HY-14649, MedChemExpress, Monmouth Junction, NJ, USA), BMS-564929 (Catalog#HY-12111, MedChemExpress, Monmouth Junction, NJ, USA), and AM580 (Cat. No.: HY-10475, MedChemExpress, Monmouth Junction, NJ, USA) were dissolved in DMSO and added to a final concentration as indicated in figures (1 μM, 1 μM, and 0.1 μM). 

### 2.3. Antibodies

Immunoblot detection, validation, and quantification experiments included primary antibodies: Rabbit Anti-POLR3G (Proteintec, Rosemont, IL, USA 24701-1-AP) [1:1000], Rabbit Anti-POLR3G (Invitrogen, Waltham, MA, USA, PA5-75727) [1:1500], Rabbit Anti-POLR3G (Invitrogen, Waltham, MA, USA, PA5-65733) [1:1500], Rabbit Anti-POLR3G (Invitrogen, Waltham, MA, USA, PA5-103799) [1:1000], Rabbit Anti-POLR3G (Invitrogen, Waltham, MA, USA, PA5-51120) [1:1000] Pol III RPC32 Antibody (C32-1) (SANTA CRUZ, sc-21754) [1:200], Pol III RPC32 Antibody (H-9) (SANTA CRUZ, sc-48365) [1:20], Rabbit Anti-POLR3GL (Novus Biologicals, NBP1-79826) [1:150], Rabbit Anti-POLR3GL (Aviva, ARP60451_P050) [1:200], Rabbit Anti-POLR3GL (Invitrogen, Waltham, MA, USA, PA5-55570) [1:150], Mouse Anti-POLR3GL (OriGene, Rockville, MD, USA, OTI5E8) [1:1000], Mouse Anti-POLR3GL (OriGene, Rockville, MD, USA, OTI5F10) [1:1000], Rabbit Anti-MYC (16286-1-AP) [1:1000], Rabbit Anti-MAX (Cell signal #4739) [1:1000], Rabbit anti ZNF131 (PA5-30641), ZNF207 Polyclonal Antibody (PA5-30641), Rabbit Anti-Lamin B2 (Cell Signaling Technologies, Danvers, Massachusetts, USA E1S1Q) [1:1000], and Rabbit Anti-TUBB (Abcam, Waltham, Boston, ab21058) [1:5000]. Secondary Goat anti-Mouse IgG (H + L) Secondary Antibody, HRP (Catalog# 31430, Invitrogen, Waltham, MA, USA) and Goat anti-Rabbit IgG (H + L) Secondary Antibody, HRP (Catalog# 31462, Invitrogen, Waltham, MA, USA) were used for immunoblot experiments. 

### 2.4. Plasmids and Transfection

ZNF131-pcDNA3.1+ and ZNF207-pcDNA3.1+ plasmids were generated using the NEBuilder^®^ HiFi DNA Assembly Master Mix (E2621S, NEB, Ipswich, MA, USA) with HEK293T cDNA using primers shown in Table 1:

pcDNA3.1+ plasmids expressing POLR3G (Catalog# OHu05486 GenScript), POLR3GL (Catalog# OHu30091, GeneScript, Piscataway, NJ, USA), MYC (Catalog# OHu27105D GenScript, Piscataway, NJ, USA), MAX (Catalog# OHu16832D GenScript, Piscataway, NJ, USA), KDM5B (Catalog# OHu64356D GenScript, Piscataway, NJ, USA), and MXD4 (Catalog# OHu05408D GenScript, Piscataway, NJ, USA) were obtained from GenScript. Plasmids for transfection were extracted with PureLink HiPure Plasmid MidiPrep (Invitrogen, Waltham, MA, USA K210005); plasmid sequences are available upon request.

MYC-siRNA (Cat #4392420) and MAX-siRNA (Cat # 4392420) were obtained from ThermoFisher, and used at a final concentration of 100 nM. In addition, the siRNA was synthesized from IDT, and the sequences and working concentrations are listed in Table 2.

HEK293T cells (2 × 10^4^ cells/cm^2^) were seeded into 6-well Nunc™ Cell-Culture Treated Multidishes (Thermo Scientific, Waltham, MA, USA, Catalog# 140675) and incubated overnight. Plasmids were transfected using Lipofectamine 3000 (Catalog# L3000001, Invitrogen, Waltham, MA, USA) according to the manufacturer’s protocol. siRNA was transfected using Lipofectamine^®^ RNAiMax (Cat # 13778150). Cells were incubated for 48 h at 37 °C with 5% CO_2_ before collection.

### 2.5. Western Blots (WB)

Cell pellets were washed once with PBS before lysis with RIPA buffer (Catalog# J62524.AD, Thermo Scientific, Waltham, MA, USA) following standard protocols. Total protein concentration was determined using a Pierce BCA protein assay kit (Catalog# 23225, Thermo Scientific, Waltham, MA, USA), equivalent protein fractions, diluted in RIPA buffer are incubated at 95℃ with diluted 4× Laemmli Sample Buffer (Catalog#1610741, BIO-RAD Hercules, CA, USA) for 5min. Proteins were separated on 4–20% Mini-PROTEAN^®^ TGX Stain-Free™ Protein Gels, 15 wells (Catalog# 4568096, BIO-RAD, Hercules, CA, USA) using 10× Tris/Glycine/SDS (Catalog# 1610732, BIO-RAD, Hercules, CA, USA) and transferred onto polyvinylidene difluoride membranes (0.2 um) (Catalog# LC2002, Invitrogen, Waltham, MA, USA) with a Trans-Blot^®^ Turbo™ Transfer System (Catalog# 1704150, BIO-RAD, Hercules, CA, USA). Transfer membranes were blocked with a 5% blotting-grade blocker (Catalog# 1706404, BIO-RAD, Hercules, CA, USA), followed by incubation with the primary antibody listed below at 4 °C overnight. Membranes were washed with TBST and incubated with mouse and rabbit secondary antibodies conjugated with horseradish peroxidase (Catalog# 31430, 31462, Invitrogen, Waltham, MA, USA) for 2 h at room temperature followed by three washes in TBST. Proteins were visualized using either SuperSignal West Pico (Catalog# 34580, Thermo Scientific, Waltham, MA, USA) or SuperSignal West Femto (Catalog# 34096, Thermo Scientific, Waltham, MA, USA) with a ChemiDoc™ Touch Imaging System (Catalog# 1708370, BIO-RAD, Hercules, CA, USA). Antibodies used for immunoblotting are listed above in the Antibodies section. Protein abundances were calculated by normalizing to reference protein Lamin B2 or β-Tubulin.

### 2.6. RNA Extraction and Real-Time Quantitative Reverse Transcription Polymerase Chain Reaction (RT−qPCR)

RNA was extracted using E.Z.N.A.^®^ Total RNA Kit I (Catalog# R6834-01, Omega, Norcross, Georgia, USA), with 100% ethanol instead of 70% at the binding step to enrich small RNA. mRNA reverse transcription was performed with 1.3–2.0 μg of RNA using the High-Capacity cDNA Reverse Transcription Kit (Catalog# 4368814, Applied Biosystems, Waltham, Massachusetts, USA). Real-time Quantitative PCR was performed with a TaqMan™ Fast Advanced Master Mix (Catalog#4444557, Applied Biosystems, Waltham, Massachusetts, USA) and predesigned TaqMan Gene Expression Assays (20×; Catalog# 4331182, ThermoFisher, Waltham, MA, USA) for selected genes: Hs02786624_g1 (GAPDH); Hs99999903_m1 (ACTB); Hs04978644_g1 (POLR3G); Hs01113209_g1 (POLR3GL); Hs00153408_m1 (MYC); Hs00811069_g1 (MAX); Hs00940446_m1 (RARA); Hs01067640_m1 (RXRA); Hs01045973_m (ZNF207); Hs00399572_m1 (ZNF131); Hs00355782_m1 (CDKN1A); Hs00153277_m1 (CDKN1B); Hs00364847_m1 (CDK4); and Hs00153380_m1 (CCND2). mRNA abundances presented were determined as the relative fold change normalized against the geometric mean of two reference genes: GAPDH + ACTB.

### 2.7. Statistical Analysis (mRNA and Protein Quantification)

Significance tests for multiple group (>2) comparisons were analyzed with a one-way analysis of variance (ANOVA) with post hoc Tukey’s HSD test. Two-group comparisons were analyzed with a *t*-test. Differences were deemed significant at *p* < 0.05. ns = not significant (*p* > 0.05); * *p* ≤ 0.05; ** *p* ≤ 0.01; *** *p* ≤ 0.001; **** *p* ≤ 0.0001. Plots were visualized with GraphPad Prism 9.3.1 (San Diego, CA, USA). 

### 2.8. Data Acquisition

Clinical signatures, which link mRNA expression and methylation patterns with TCGA patient outcome data, were retrieved from tcga-survival.com (accessed on 29 October 2022) and correspond to cancer-specific and summary z-scores calculated using Cox univariate hazard models [19]. TCGA RNA-seq data were retrieved across all TCGA cancer types from the Broad Institute TCGA Genome Data analysis Center (GDAC) Firehose mRNASeq Level 3 RSEM gene normalized data files available at https://gdac.broadinstitute.org/ (accessed on 29 March 2022) [20]. Primary solid tumor ATAC-seq alignment bam files were previously retrieved from the Genomic Data Commons Data Portal (https://portal.gdc.cancer.gov (accessed on 1 January 2021)) [21]. Gene expression dynamics related to ATRA and other bioactive molecules were obtained from the Signaling Pathways Project (http://www.signalingpathways.org (accessed on 31 May 2023)) [22]. Data were specifically subset for *POLR3G*-specific fold changes across all datasets, and ranked according to -log_10_(adj, p-val) × log_2_(fold change). Multi-context gene expression survey data were obtained from ProteinAtlas (https://www.proteinatlas.org/about/download (accessed on 30 May 2022)) [23]. ChIP-seq peak overlap, ChIP-seq signal files, bisulfite-sequencing data, and transcription factor target gene sets were obtained from ChIP-atlas (https://github.com/inutano/chip-atlas/wiki#downloads_doc (accessed on 6 June 2023)) [24]. Composite ChIP-seq data tracks were generated by taking the upper quartile signal value across all available ChIP signal files unless otherwise noted. Intact Hi−C loop calls were obtained from ENCODE (https://www.encodeproject.org/ (accessed on 3 August 2023)) [25]. Human Super-Enhancer coordinates were obtained from SEdb2.0 (https://bio.liclab.net/sedb/ (accessed on 25 July 2023)) [26]. 

### 2.9. Pan-Cancer Co-Expression and Chromatin Accessibility Correlation Analyses

Intra-cancer gene (co-expression) correlations were determined with Spearman’s rank method implemented across all the genes. Z-transformation of gene correlation scores was defined as the difference between the observed and the mean correlation divided by the standard deviation for each gene. Pan-cancer gene correlation analyses were determined by calculating the median correlation value for every gene across the distribution of cancer subtypes.

Chromatin accessibility correlation scores were determined with Spearman’s rank method. Genomic coordinates corresponding to a 1 Mb window centered on *POLR3G* (chr5: 89,970,000–91,020,000) were first divided into 100 bp bins. TCGA ATAC-seq read counts were extracted across individual bins, as well as a reference list of ~200,000 additional coordinates for global normalization. ATAC-seq counts corresponding to all 409 TCGA tumor samples were quantile normalized and integrated with RNA-seq data using matched sample IDs. Intra-sample correlations between *POLR3G* expression and normalized ATAC values were determined with Spearman’s rank method. Correlation *p*-values were adjusted using the Benjamini–Hochberg method, and subsequent analyses were subset on bins with adjusted *p*-values < 0.0001 unless otherwise noted. 

### 2.10. Overlap Enrichment Analyses (TFs, DNA-Methylation, and ATAC)

Overlap events were mapped to genomic coordinates corresponding to 100 bp bins with significant correlation values (see above) using the ChIP-Atlas Enrichment Analysis tool (https://chip-atlas.org/enrichment_analysis) [24]. Observed and expected overlap frequencies for each experiment were first retrieved using 100 permutations and a q-value cutoff of < 1 × 10^−5^. Enrichment for each individual factor was then assessed in relation to the full distribution of relevant factors (e.g., ChIP (histone or TF and other factors), bisulfite-seq (sample/context), or ATAC-seq (sample/context)). Briefly, expected (random) overlap frequencies were subtracted from observed overlap frequencies for each individual experiment, generating an “observation beyond expectation” overlap number for each experiment, which accounts for the number of experiment-specific binding events. For each unique feature, an aggregate observation beyond the expectation total was then compared to a non-parametric distribution with a permutation test (*n* = 10,000), controlling for the same number of experiments. The enrichment of each feature was assessed by multiplying the log_2_(observed/expected overlap frequency) by −log_10_(adj. *p*-value), plotted against the number of standard deviations beyond expectation (z-score). Bins were integrated with all available loops’ calls mapped with intact Hi−C across 72 tissues [25] and colored on the basis of intersection between negative bins and DNA loop anchors.

### 2.11. Gene Ontology (GO) and Gene Set Enrichment Analysis (GSEA)

Both GO and GSEA enrichment analyses were performed on lists of TF target genes, which were retrieved from ChIP-atlas (e.g., https://chip-atlas.dbcls.jp/data/hg38/target/ (accessed on 6 June 2023) MYC.1.tsv) and defined as having TF occupancy within 1 Kb of a given gene promoter. TF target genes were post-filtered with a required minimum average MACS2 score > 50. GO (cellular compartment, “CC”) ontology enrichment was then determined using clusterProfiler [27], performed on TF target gene lists in relation to a gene “universe” corresponding to all unique genes targeted by any TF mapped with ChIP-atlas. GSEA analyses were performed on genes ranked with pan-cancer *POLR3G* correlation scores (median z-score) using the fgsea package in R (https://github.com/ctlab/fgsea (accessed on 28 February 2023)), setting nPermSimple = 10,000. The normalized enrichment score (NES) and adjusted *p*-value for each TF target gene set were then compared against all TF target gene sets. 

### 2.12. Gene- and TF-Distance Analyses

TF target gene lists, defined with the presence of a given TF within ± 1 Kb of a TSS, were retrieved from ChIP-atlas as described. A score matrix, defined with the mean MACS2 score for each TF–gene pair, was converted to a binary matrix representing presence (score > 50) or absence (score < 50) of each TF. The Euclidean distance between *POLR3G* and each gene was calculated with respect to the binary score for all TFs. Dimension reduction with a principal component analysis (PCA) was performed after removing genes with 0 variance and individual genes were projected on PC1 and PC2 axes. Genes were then binned with ranked distance as a proxy for regulatory similarity. PCA was analogously performed on individual TFs on the basis of the presence of each individual gene in a given TF target list.

## 3. Results

### 3.1. The Gene Encoding RPC7 Subunit RPC7α Is a Distinctively Negative Prognostic Factor in Multiple Cancer Subtypes

Subunits RPC7α and RPC7β give rise to two forms of the RPC3-6-7 subcomplex and, in this way, underlie two distinct Pol III identities (Figure 1a,b). Recent Cryo-EM structural analyses of the full human Pol III complex have resolved several protein–protein interfaces of subunit RPC7α, including site-specific interactions with the stalk module, N-terminus interactions with the clamp domain, as well as insertion of the RPC7α C-terminus into the DNA channel and active site of Pol III transcription [6,28,29,30]. The RPC7 subunits share 46% amino acid identity [7], with highly conserved regions dispersed throughout the primary sequence, including regions mapping to the stalk interface and C-termini [31]. Given the sequence similarity of RPC7α and RPC7β, we screened multiple antibodies for subunit-specific specificity. Ectopic expression and an immunoblot analysis of flag-tagged RPC7α and RPC7β subunits in HEK293T confirm subunit-specific detection and distinguish antibodies with specificity from reagents that exhibit variable levels of cross-reactivity (Figure 1b and Figure S1).

Multiple reports have linked high *POLR3G* (RPC7α) expression with unfavorable outcomes across distinct cancer subtypes [14,15,16,17]; however, few studies have directly compared clinical signatures associated with *POLR3G*, *POLR3GL,* or other Pol III subunits. Here, we profiled the relationship between *POLR3G* (RPC7α) or *POLR3GL* (RPC7β) expression and outcomes among cancer patients, stratified by cancer subtype (Figure 1c). We specifically explored univariate Cox proportional hazard models reported by Smith and Sheltzer [19], in which the relationship between mRNA abundance and patient outcomes was quantified using gene expression profiles generated across all TCGA (The Cancer Genome Atlas) cancer subtypes. Cox models are well suited for continuous data, obviating the need for threshold selection and dichotomization of gene expression data. Cancer-specific and pan-cancer z-scores reflect the directionality and significance of survival relationships for individual genes [19]. Consistent with previous findings, *POLR3G* expression is commonly associated with unfavorable outcomes, most notably in clear cell renal cell carcinoma (KIRC) and brain lower-grade glioma (LGG). In contrast, *POLR3GL* expression is rarely unfavorable and is instead associated with favorable outcomes in multiple cancers. This phenomenon is flipped for *POLR3G* and *POLR3GL* in acute myeloid leukemia (LAML), however, suggesting a potentially unique role of RPC7α and RPC7β in myeloid cells with implications for disease progression (Figure 1c). Altogether, *POLR3G* is most significantly associated with unfavorable outcomes in pan-cancer analyses compared to all other Pol III subunits, residing in the top 3.39% of unfavorable genes (Figure S2), further highlighting the need to understand the regulation and function of *POLR3G*.

### 3.2. An Integrated Survey of POLR3G mRNA and Chromatin Correlates in Cancer Identifies Candidate Regulatory Factors and Sequence Elements

We analyzed mRNA and chromatin accessibility signatures captured with RNA-seq and ATAC-seq, respectively, to predict regulatory factors and elements involved in *POLR3G* upregulation across TCGA cancer subtypes [20]. We reasoned that transcription factors that enhance or repress *POLR3G* are likely to correlate with *POLR3G* mRNA levels. Similarly, chromatin accessibility at regulatory elements involved in *POLR3G* regulation should also correlate with *POLR3G* mRNA levels. In this way, connecting co-expression correlation (Figure 1d) with dynamic chromatin correlates (Figure 1e) provides an unbiased approach to identify candidate regulators of *POLR3G* expression. *POLR3G* expression may also be determined with signal-activated factors and thus, in the absence of co-expression correlation, enrichment of specific transcription factor binding patterns at dynamic chromatin signatures may also identify important players in *POLR3G* regulation (Figure 1e).

Our co-expression survey first considered all cancer subtypes individually by determining correlation scores between *POLR3G* and all other genes in each cancer. Correlation scores were then transformed to a standardized z-score and, finally, a median z-score was computed for each feature (Figure 1d). Thus, a high or low median z-score is a more confident indicator and signifies a strong association with *POLR3G* expression across most cancers. We find that MYC has a relatively high median z-score, suggesting MYC levels may be broadly important for *POLR3G* expression in cancer (Figure 1f). OCT4, NANOG, KLF4, and SOX2, on the other hand, are not associated with *POLR3G* expression, suggesting a limited or subtype-restricted role for pluripotency factors in cancer. Notably, several factors with high or low median z-scores are identified as candidate regulators in our co-expression survey. ZNF131, for example, is the strongest DNA-binding transcription factor positively linked with *POLR3G* mRNA abundance in cancer, whereas MAX-dimerization protein 4 (MXD4) is the strongest negative correlate (Figure 1f). Among the list of co-expressed features of interest, both RARA (retinoic acid receptor alpha) and RXRA (retinoid X receptor alpha) are identified as strong negative correlates, whereas ZNF207, an early developmental factor linked with pluripotency factors [33], is a strong positive correlate in cancer (Figure 1f).

Our survey of *POLR3G*-chromatin correlation scores, on the other hand, connects genome-wide accessibility profiles in 409 tumor samples to integrated mRNA profiles in matched samples. Correlations were determined between each 100 bp bin within 500 Kb (1 Mb window) of *POLR3G* with the corresponding level of mRNA abundance in matched samples. Genomic coordinates with significant FDR scores (<0.0001) were retained as either positive or negative DNA coordinates and candidate *POLR3G*-related regulatory elements (Figure 1e). We find that genomic coordinates proximal to but downstream of the *POLR3G* transcription start site are the strongest positive correlates with *POLR3G* expression, whereas the strongest negative correlates are identified ~400 Kb downstream (Figure 1g). Taken together, the co-expression and chromatin accessibility surveys identify candidate regulatory factors and genomic coordinates of interest related to *POLR3G* upregulation in cancer. We therefore next sought to integrate the DNA-binding profiles mapped for specific positive and negative factors with predicted regulatory sequences. 

### 3.3. A Gene-Internal Element, Corresponding to an Early Developmental Super-Enhancer, Is the Strongest Chromatin Correlate with POLR3G Expression in Cancer

**Figure 2:**
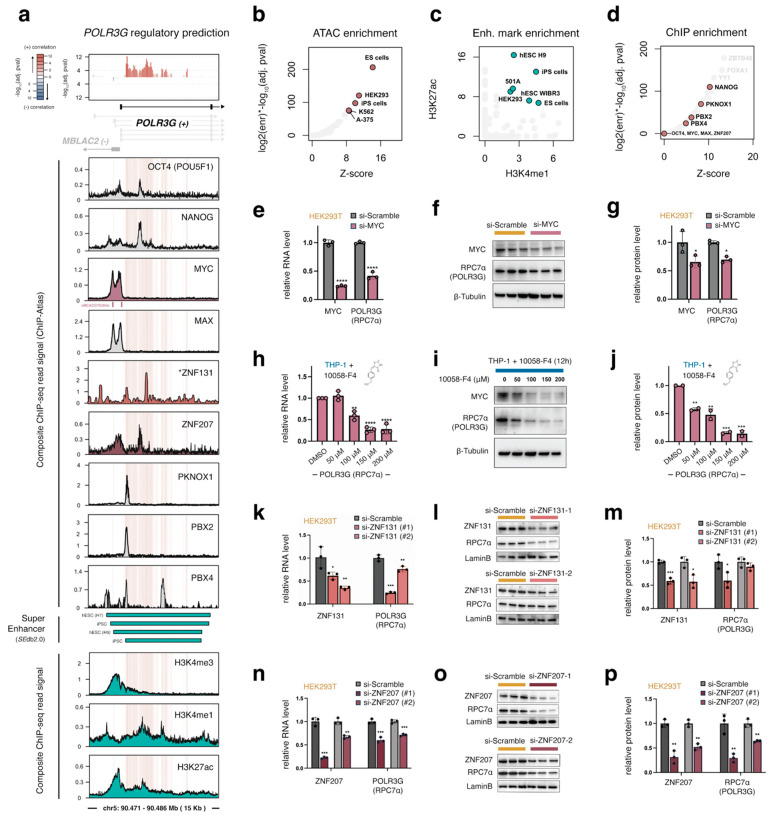
A multimodal regulatory platform, including a gene-internal element linked to multiple transcription factors, promotes *POLR3G* expression and RPC7α abundance. (**a**) ChIP-seq profiles for known and candidate positive regulators of *POLR3G* expression, including pluripotency factors (OCT4 and NANOG), MYC/MAX, ZNF131, and ZNF207, overlayed on the *POLR3G* gene promoter and DNA bins with significant correlation scores (top). Height and shading denote the significance (−log_10_(adj. pval)) of positive (red) and negative (blue) chromatin correlation scores at each 100 bp bin. Gene track includes the dominantly expressed *POLR3G* isoform (black); alternate *POLR3G* transcript annotations (light gray); and *MBLAC2* (dark gray). + strand orientation (right arrow) and − strand orientation (left arrow) shown. * All ChIP-seq profiles shown are composite signals derived from ChIP-atlas, with the exception of ZNF131 (ZBTB35 ChIP data in HEK293 cells [35]). Additional signals shown for TFs (PKNOX1, PBX2, and PBX4), histone marks (H3K4me3, H3K4me1, and H3K27ac), and annotated super enhancers (SEdb2.0) [26]. (**b**) Context-specific enrichment for ATAC peak overlap with DNA coordinates featuring significant positive correlation with *POLR3G* expression (padj < 0.0001). (**c**) Context-specific enrichment for H3K4me1 (*x*-axis) and H3K27ac (*y*-axis) peak overlap with DNA coordinates featuring significant positive correlation with *POLR3G* expression (padj < 0.0001). Contexts with both high H3K4me1 and H3K27ac are highlighted. (**d**) Enrichment for ChIP-seq peak (TFs and other factors) overlap with DNA coordinates featuring significant positive correlation with *POLR3G* expression (padj < 0.0001). (**e**) Relative mRNA abundance of *MYC* and *POLR3G* (RPC7α) after siRNA mediated *MYC* knockdown in HEK293T. (**f**,**g**) Immunoblots and quantification of MYC, POLR3G (RPC7α), and β-Tubulin protein levels following MYC knockdown. (**h**) Dose–response RT−qPCR analysis of *POLR3G* (RPC7α) mRNA level following exposure of THP−1 monocytes to 10058-F4 (F4). (**i**) Immunoblot detection of MYC, RPC7α (*POLR3G*), and β-Tubulin protein abundance following F4 exposure. (**j**) Relative protein abundance of RPC7α (*POLR3G*) after F4 treatment. (**k**) RT−qPCR analysis of *ZNF131* and *POLR3G* mRNA levels following siRNA-mediated *ZNF131* knockdown. (**l**,**m**) Immunoblots and quantification of ZNF131 and POLR3G (RPC7α) protein levels following siRNA-mediated *ZNF131* knockdown. (**n**) RT−qPCR analysis of *ZNF207* and *POLR3G* mRNA level following siRNA-mediated *ZNF207* knockdown. (**o**,**p**) Immunoblots and quantification of ZNF207 and POLR3G (RPC7α) protein levels following siRNA-mediated *ZNF131* knockdown. Biological replicates = 3; two-group comparison (**e**,**g**) analyzed with *t*-test; multi-group comparison (**h**,**j**) analyzed with ANOVA; * *p* ≤ 0.05; ** *p* ≤ 0.01; *** *p* ≤ 0.001; **** *p* ≤ 0.0001.

Our chromatin correlation survey indicates that DNA accessibility downstream of the *POLR3G* transcription start site is the most significant predictor of *POLR3G* expression surveyed across 409 tumor samples (Figure 1g and Figure 2a). TF occupancy profiles for OCT4, NANOG, and MYC, which have previously been linked to *POLR3G* expression [18], reveal binding of pluripotency factors OCT4 and NANOG within this predicted regulatory element, whereas MYC and MAX occupancies are restricted to the *POLR3G* gene promoter, overlapping two E-box target sequences (CACGTG) (Figure 2a), which in eukaryotes facilitates protein-DNA binding [34]. Notably, we find that the gene-internal element maps to an early-developmental super-enhancer. Accordantly, this internal enhancer element is enriched for chromatin accessibility (ATAC-seq; Figure 2b) and active histone marks, H3K4me1 and H3K27ac in ES and iPSCs (Figure 2c). HEK293 cells show similarly high DNA accessibility and active enhancer mark enrichment at this element, and we confirm with expression and protein abundance surveys that HEK293T cells express comparatively high levels of RPC7α when compared to nonembryonic human cell lines (Figure S3).

An overlap enrichment analysis of >1000 factors uniformly mapped with ChIP-atlas [24] further indicates that a multitude of candidate regulatory factors target the *POLR3G* gene-internal element. For example, enrichment scores (which, we note, are influenced by ChIP and peak quality, binding frequencies, and experimental replicates) link NANOG and several other TFs to DNA bins with significant positive correlation (Figure 2d). We find that specific factors, such as PKNOX1, PBX2, and PBX4, appear to target subregions with particularly high correlation scores with *POLR3G* expression (Figure 2a,d). Overall, our chromatin survey points to the gene-internal element and its status as a putative super-enhancer as a strong predictor of *POLR3G* mRNA abundance, and altogether suggests that a re-commissioned super-enhancer enriched for multiple factors may be a central determinant of *POLR3G* expression in cancer.

### 3.4. MYC Promotes POLR3G Expression Independently of the Gene-Internal Regulatory Element

In contrast to the gene-internal regulatory element, accessibility of the *POLR3G* gene promoter does not correlate with *POLR3G* expression in cancer. This finding implies that transcription factor recruitment to the *POLR3G* promoter is not a major determinant of *POLR3G* expression, or that accessibility is instead maintained in the absence of *POLR3G* transcription. We therefore sought to further examine the importance of MYC, which targets the *POLR3G* promoter but does not localize to the gene-internal element. Indeed, we find that knockdown of MYC in HEK293T results in significant downregulation of *POLR3G* expression and RPC7α protein abundance (Figure 2e–g), whereas MYC overexpression increases both *POLR3G* and RPC7α levels (Figure S4). We further demonstrate that inhibition of MYC/MAX dimerization, which also significantly reduces MYC protein levels, effectively disrupts *POLR3G* expression in THP−1 monocytes (Figure 2h–j). 10058-F4, a small molecule inhibitor of MYC/MAX dimerization in myeloid cells [36], also disrupts *CCND2* and *CDK4* expression and inversely increases *CDKN1A* and *CDKN1B* levels—canonical MYC targets—in THP−1 (Figure S5) [37]. These effects are mirrored by dose-dependent decreases in *POLR3G* mRNA and RPC7α protein abundance (Figure 2h–j), confirming MYC as an important mediator of *POLR3G* expression, independent from, and potentially in conjunction with, the gene-internal regulatory platform identified in our chromatin accessibility survey.

### 3.5. Identification of Zinc Finger Proteins ZNF131 (ZBTB35) and ZNF207 (BuGZ) as Additional Regulatory Factors That Promote POLR3G Expression

Beyond MYC, our *POLR3G* co-expression correlation survey identifies specific transcription and chromatin-related factors with even stronger correlation signatures in cancer. In particular, the expression of *ZNF131* (also referred to as ZBTB35) is the strongest positive predictor of *POLR3G* expression among transcription factors (Figure 1f). Available ZNF131 (ZBTB35) ChIP-seq data in HEK293 cells feature some level of signal enrichment within the *POLR3G* gene-internal regulatory element identified in our study (Figure 2a). ZNF131 was previously shown to be important in the growth and development of multiple tissues [34,38]. In addition to ZNF131, we find that ZNF207, a transcription factor previously linked to OCT4 and other pluripotency factors and a significant correlate with *POLR3G* expression (Figure 1f) [33], is similarly characterized by a ChIP-seq signal over the gene-internal element as well as the *POLR3G* gene promoter (Figure 2a). We therefore tested the effect of ZNF131 and ZNF207 perturbations on *POLR3G* expression in HEK293T cells. As shown for MYC, knockdown of either ZNF131 or ZNF207 results in significant loss of *POLR3G* mRNA and RPC7α protein abundance (Figure 2k–p), whereas overexpression of ZNF131 or ZNF207 enhances *POLR3G* expression, even in a context with comparatively high *POLR3G* mRNA abundance (Figure S6). These data demonstrate that multiple factors associated with *POLR3G* occupancy and co-expression correlation in cancer functionally enhance *POLR3G* expression, suggesting *POLR3G* gene activity may be controlled in a combinatorial and/or additive manner with gene-activating transcription factors.

### 3.6. Gain of DNA Methylation over the Gene-Internal Regulatory Element Coincides with Developmental Loss of POLR3G Expression

**Figure 3:**
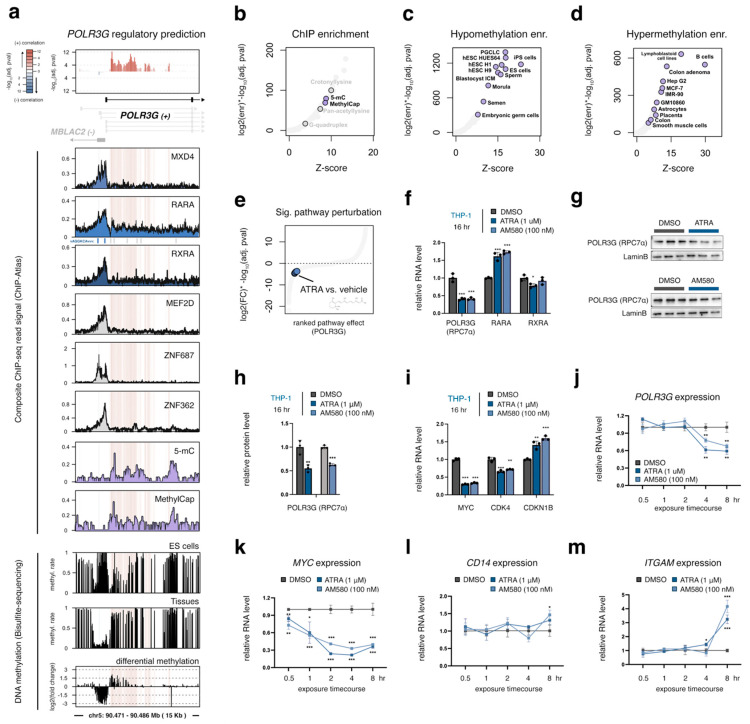
*POLR3G* expression is negatively associated with gene-internal DNA methylation and rapidly downregulated in response to Retinoic Acid. (**a**) ChIP-seq profiles for candidate negative regulators of *POLR3G* expression, including MXD4, Retinoic Acid Receptor Alpha (RARA), Retinoid X Receptor Alpha (RXRA), MEF2D, ZNF687, and ZNF362, overlayed on the *POLR3G* gene promoter and DNA bins with significant positive correlation scores (top). DNA-methylation profiles shown include IP experiments against 5-methylcytosine (5-mC; MethylCap), and methylation rates determined with bisulfite sequencing in embryonic stem (ES) cells and tissues. (**b**) Enrichment for ChIP-seq peak (TFs and other factors) overlap with DNA coordinates featuring significant positive correlation with *POLR3G* expression (padj < 0.0001), highlighting experiments targeting chemical modification. (**c**,**d**) Context-specific enrichment for DNA coordinates, annotated as hypomethylated (**c**) or hypermethylated (**d**) with bisulfite sequencing, with DNA bins featuring significant positive correlation with *POLR3G* expression (padj < 0.0001). (**e**) *POLR3G* gene expression fold change scores: log_2_(fold change over control) × –log_10_(adjusted *p*-value) in response to bioactive molecule treatment, dots represent individual experiments, ATRA vs. vehicle highlighted in blue, and data obtained from http://www.signalingpathways.org (accessed on 31 May 2023). (**f**) RT−qPCR analysis of *POLR3G*, *RARA*, and *RXRA* mRNA levels in response to ATRA or AM580 treatment. (**g**,**h**) Immunoblots and quantification of POLR3G (RPC7α) protein levels following ATRA or AM580 treatment. (**i**) RT−qPCR analysis of *MYC* and MYC target genes *CDK4* and *CDKN1B* mRNA levels in response to ATRA or AM580 treatment. (**j**–**m**) RT−qPCR analysis of *POLR3G*, *MYC*, and macrophage differentiation markers *CD14* and *ITGAM* expression at 0.5, 1, 2, 4, and 8 h after ATRA or AM580 treatment. mRNA levels are normalized with DMSO (vehicle) group at individual time points. (**f**–**m**) Biological replicates = 3; * *p* ≤ 0.05; ** *p* ≤ 0.01; *** *p* ≤ 0.001.

The upregulation of *POLR3G* expression in cancer may be related to changes in either activating and/or repressive mechanisms. We therefore re-examined the *POLR3G* promoter and gene-internal regulatory element in relation to candidate negative regulatory factors and established silencing mechanisms. A survey of transcription factors negatively linked with *POLR3G* expression uncovers variable levels of ChIP-seq signal enrichment at the *POLR3G* gene promoter, but not within the gene-internal element (Figure 3a). Immunoprecipitation (IP) experiments against DNA methylation, however, are enriched at bins mapping to the internal regulatory element (Figure 3a,b), and bisulfite sequencing experiments indicate particularly high methylation rates within this element in specialized tissues (Figure 3a). DNA methylation levels specifically increase within the gene-internal regulatory element in tissues compared to ES cells (Figure 3a), whereas ES cells are instead enriched for hypomethylation (Figure 3c). We find that early developmental contexts, including the blastocyst inner cell mass (ICM), the morula, and primordial germ-like cells (PGCLC), are similarly enriched for hypomethylation across the gene-internal elements (Figure 3c), whereas a variety of differentiated cell types and cell lines are enriched for hypermethylation (Figure 3d). These data indicate that the regulatory platform targeted by pluripotency factors and other TFs may be decommissioned with deposition or maintenance of DNA methylation patterns. We also note that, in contrast to high *POLR3G* expression, methylation of *POLR3G* is a weakly positive prognostic feature (Figure S7), altogether suggesting methylation may play an important role in diminishing the ability of the gene-internal element to recruit transcription factors that increase *POLR3G* expression in cancer. 

### 3.7. POLR3G mRNA Levels Decrease Early in Response to Retinoic Acid, but Subsequent to MYC Downregulation and Concomitant with Markers of Differentiation

Retinoic acid receptor alpha (RARA) and retinoid X receptor alpha (RXRA), which negatively correlate with *POLR3G* expression across cancers, are retinoid-activated nuclear receptors that mediate transcriptional programs by forming homo- and heterodimers in the presence or absence of specific ligands [39]. As transcription factors, these proteins target specific response elements typically characterized by two, repeated 6 bp sequences. The distance separating each target element can play an important role in shaping whether RAR/RXR heterodimers function as transcriptional activators or repressors, with effects potentiated by ligand activation [40]. In the case of *POLR3G*, a candidate retinoic acid response element (AGGTCA[]CGGTCT) is located upstream of the transcription start site (Figure 3a). This element is a putative “DR0”, a direct repeat separated with a 0-nt spacer, the most prevalent sequence example identified in RAR/RXR studies in mice [41].

In human embryonic stem cells (hESCs), retinoic acid (RA) is known to induce differentiation and loss of *POLR3G* expression, though a direct role in *POLR3G* regulation has not been reported [42]. A survey of *POLR3G* dynamics mapped with the Signaling Pathways Project, which integrates thousands of gene expression experiments in response to bioreactive molecules and signaling pathways, identifies a significant loss of *POLR3G* expression in NTERA-2 cells in response to All-trans Retinoic Acid (ATRA), a ligand that activates both RAR and RXR factors (Figure 3e) [22,43]. In THP−1 monocytes, which express both *RARA* and *RXRA* (Figure S8), we find that exposure to either the general RAR:RXR ligand, ATRA, or selective RARA agonist, AM580, induces significant upregulation of *RARA*, as well as downregulation of *POLR3G* mRNA and RPC7α protein levels at 16 h post-exposure (Figure 3f–h). However, ATRA is also known to drive sharp decreases in *MYC* expression [44], and we find that *MYC* and canonical MYC targets *CDK4* and *CDKN1B* are down- and upregulated, respectively, demonstrating that RARA and RXRA agonists induce a dynamic MYC transcriptional program in THP−1 monocytes within 16 h (Figure 3i). Longitudinal profiling of *MYC* and *POLR3G* mRNA abundance further shows that *MYC* expression is rapidly downregulated within 30 min, preceding *POLR3G* downregulation at ~4 h post-exposure (Figure 3j,k). We find that markers of monocyte-to-macrophage differentiation, including *CD14* and *ITGAM*, begin to increase between 4 and 8 h (Figure 3l,m). While RARA and RXRA may chronologically downregulate *POLR3G* following rapid changes in MYC and other factors, these findings do not rule out a potential direct role for RARA/RXRA in the delayed silencing of *POLR3G* expression.

### 3.8. MXD4, the Strongest Negative POLR3G Correlate, Limits POLR3G Expression

**Figure 4:**
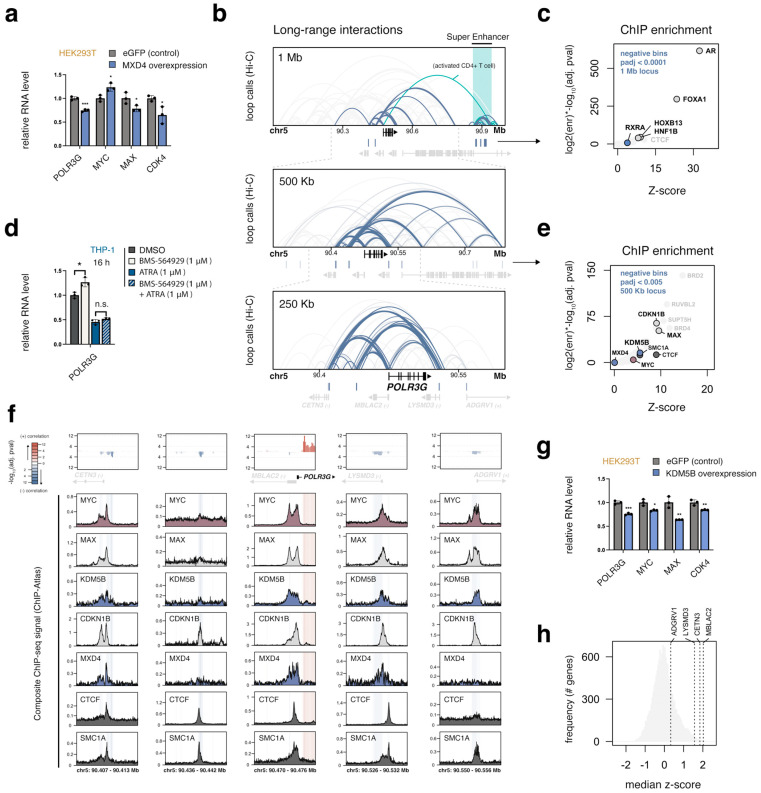
*POLR3G* is downregulated by Max-dimerization protein 4 (MXD4) and negatively linked to a local multi-promoter architectural hub enriched for MAX, CDKN1B, and KDM5B. (**a**) RT−qPCR analysis of *POLR3G*, *MYC*, *MAX*, and *CDK4* mRNA abundance following overexpression of eGFP (control) or MXD4. (**b**) Multi-tissue profile of long-range interactions mapped with intact Hi−C (ENCODE) [25], centered on *POLR3G*. DNA loops intersecting DNA bins with significant negative correlation scores are highlighted in blue. Top: 1 Mb window; most significant bins correspond to a distal super-enhancer highlighted in green. Middle: 500 Kb window; negative bins are highlighted with a relaxed significance cutoff (padj < 0.005). Bottom: 250 Kb window; a local multi-promoter hub connects *POLR3G* and neighboring genes (*CETN3*, *MBLAC2*, *LYSMD3*, and *ADGRV1*). (**c**) Enrichment for ChIP-seq peak (TFs and other factors) overlap with DNA coordinates featuring significant negative correlation with *POLR3G* expression (padj < 0.0001). (**d**) RT−qPCR analysis of *POLR3G* mRNA levels in response to AR agonist BMS-564929, RARA/RXRA agonist ATRA, or combined exposure to both ligands. (**e**) Enrichment analysis repeated for DNA coordinates featuring significant negative correlation with *POLR3G* expression (padj < 0.005), restricted to a 500 Kb window. (**f**) Individual profiles of DNA bin correlation scores, gene annotation, and specific ChIP-seq signal for factors enriched for overlap with negative bins. (**g**) RT−qPCR analysis of *POLR3G*, *MYC*, *MAX*, and *CDK4* mRNA abundance following overexpression of eGFP (control) or KDM5B. (**h**) *POLR3G* neighboring genes, *CETN3*, *MBLAC2*, *LYSMD3*, and *ADGRV1*, positively correlate with *POLR3G* expression in cancer. Distribution of *POLR3G* pan-cancer median z-scores for all genes, highlighting multi-promoter hub genes *CETN3*, *MBLAC2*, *LYSMD3*, and *ADGRV1*. Biological replicates = 3; * *p* ≤ 0.05; ** *p* ≤ 0.01; *** *p* ≤ 0.001.

Given that RA-induced *POLR3G* dynamics may be deeply rooted in multiple differentiation-related transcriptional programs, we next turned our attention to MXD4, the strongest negative TF correlate identified in our co-expression correlation survey (Figure 1f). MXD4 is a MAD family protein and putative tumor suppressor that, through heterodimerization with MAX, antagonizes MYC and downstream gene regulatory activities [45]. Consistent with its role as a transcriptional repressor, we show that overexpression of MXD4 in HEK293T is sufficient to reduce *POLR3G* expression (Figure 4a), confirming a functional role for MXD4 in *POLR3G* regulation. MXD4 overexpression does not reduce MYC expression, further demonstrating that, in contrast to retinoic acid, the interplay of MXD4 and *POLR3G* expression is not a consequence of reduced MYC availability (Figure 4a). We also note that *MXD4* expression is a significantly favorable prognostic factor, including in comparison to all 108 basic helix−loop−helix (bHLH)−containing transcription factors (Figure S10). These data indicate an important role for MXD4 in shaping *POLR3G* transcription and further highlight the clinical significance of MXD4 in cancer.

### 3.9. A Local Multi-Promoter Hub Enriched for MAX, CDKN1B, and KDM5B Is Negatively Linked to POLR3G Expression

Our pan-cancer chromatin survey identifies a broad region of significant negative correlation approximately 400 Kb downstream of *POLR3G* (Figure 1g and Figure S11). We find that the genomic coordinates mapping to these sites correspond to a multi-tissue super-enhancer enriched for the androgen receptor (AR), pioneer factor FOXA1, RXRA, and other factors (Figure 4b,c). However, long-range interaction data derived from 72 tissues identify only a single instance of an enhancer–promoter interaction between this element and *POLR3G*, restricted to activated CD+ T cells (Figure 4b). Exposure of THP−1 cells to BMS-564929, a highly potent and selective AR agonist [46], increases rather than decreases *POLR3G* expression, suggesting that AR may be involved in *POLR3G* upregulation rather than downregulation in response to ligand activation (Figure 4d). Simultaneous exposure of THP−1 monocytes to both retinoic acid and the androgen receptor agonist does not diminish retinoic-acid-induced *POLR3G* downregulation, however, potentially discounting an AR-RARA competition model for *POLR3G* regulation.

The distal super-enhancer identified in our chromatin survey, which appears as broadly active across multiple tissues, may primarily relate to differentiation status rather than direct *POLR3G* regulation. We therefore focused on proximal negative chromatin correlates and re-visited transcription factor overlap enrichment with relaxed significance thresholds (padj. < 0.005). In contrast to the distal super-enhancer, we find that negative chromatin correlates within 500 Kb map to elements related to long-range *POLR3G* promoter loops across a multitude of contexts (Figure 4b). Significant negative bins specifically correspond to promoter-proximal elements in *POLR3G*-neighborhing genes, including *CETN3*, *MBLAC2*, *LYSMD3*, and *ADGRV1* (*GPR98*), and are enriched for BRD2/4, RUVBL2, CTCF, and cohesin complex subunits involved in long-range interactions (Figure 4e,f) [47]. Notably, we find that MAX and CDKN1B (p27) are also highly enriched across the local negative bins, as well as KDM5B—an H3K4 histone demethylase that functionally represses target genes [48]. 

Like MXD4, we find that overexpression of KDM5B similarly restricts *POLR3G* expression, albeit modestly (Figure 4g). KDM5B overexpression also reduces both *MYC* and *MAX* mRNA levels, as well as MYC-target gene *CDK4*, suggesting KDM5B levels may broadly reduce transcription or otherwise similarly antagonize *MYC* expression. Nevertheless, KDM5B enrichment across the multi-promoter hub also suggests that KDM5B and other factors may coordinately regulate the expression of *POLR3G* with neighboring genes. In support of this model, we find that *CETN3*, *MBLAC2*, and *LYSMD3*, core members of the *POLR3G* promoter hub, feature particularly high correlation z-scores across cancers (Figure 4h). *ADGRV1*, which displays far less promoter connectivity, has a weak but positive association with *POLR3G* expression. The observed relationship between *POLR3G* and neighboring genes is far greater than expected with random chance, and *POLR3G* is a uniquely negative prognostic factor among its cohort, demonstrating that connections drawn between *POLR3G* and clinical outcome signatures are not a simple reflection of coordinate gene regulation (Figure S12). 

### 3.10. MYC, MAX, MXD4, and KDM5B Target an Overlapping Set of Genes Important for Cell Growth, including Most RNA Polymerase Subunits

**Figure 5:**
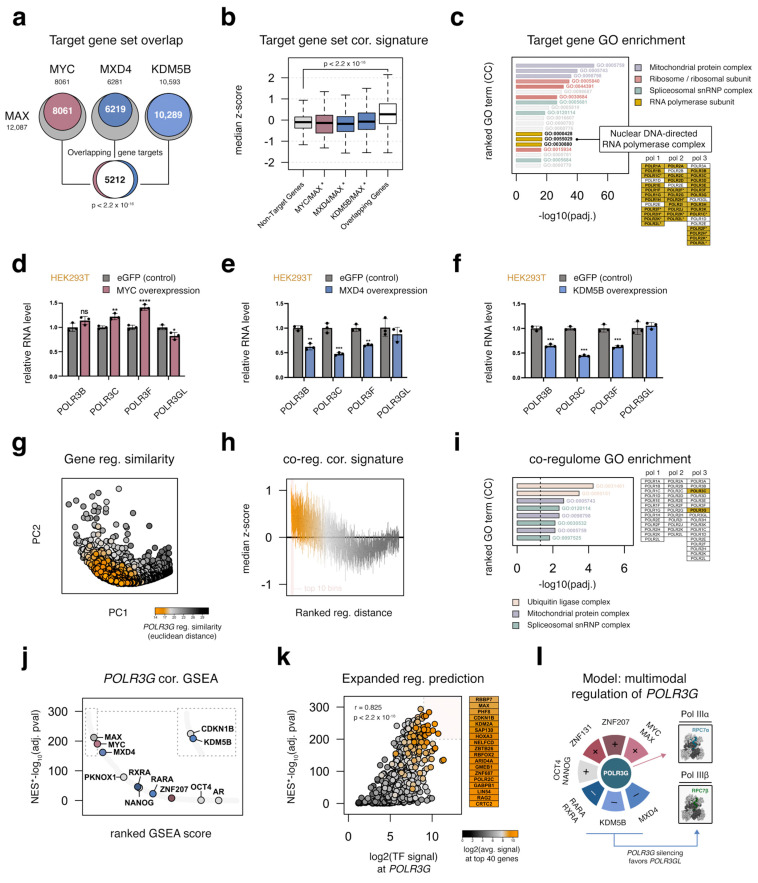
Combinatorial *POLR3G* regulation includes the convergence of factors targeting genes broadly involved in RNA processing, protein synthesis, and mitochondrial function. (**a**) Overlap comparison of target gene sets for MAX with MYC, MXD4, and KDM5B. (**b**) Comparison of pan-cancer *POLR3G* correlation score (median z-score) distributions for shared MYC/MAX target genes, shared MXD4/MAX target genes, shared KDM5B/MAX target genes, and the fully overlapping MYC/MAX/MXD4/KDM5B target gene set with all non-target genes. (* denotes that genes shared by all 4 factors are removed from doublet gene sets). (**c**) Cellular Compartment (CC) Gene Ontology (GO) enrichment analysis for overlapping target genes; GO terms ranked by −log_10_(FDR). GO terms related to RNA polymerase subunits are highlighted in gold. Individual genes encoding all RNA polymerase I, II, and III subunits are presented—genes that are included in the overlapping target gene set are highlighted in gold. (**d**–**f**) RT−qPCR analysis of mRNA abundance related to genes encoding RNA polymerase III subunits RPC2 (*POLR3B*), RPC3 (*POLR3C*), RPC6 (*POLR3F*), and RPC7β (*POLR3GL*) following overexpression of MYC, MXD4, or KDM5B in HEK293T cells compared with eGFP (pcDNA3.1; control). (**g**) Visualization of gene regulatory similarity, defined with the presence or absence of 1662 factors across all gene promotors (1 Kb). Color depicts Euclidean distance between each gene and *POLR3G*, projected onto coordinates for principal components PC1 and PC2. (**h**) Comparison of pan-cancer *POLR3G* correlation score (median z-score) distributions as a function of *POLR3G* distance (as defined in Figure 5d). Boxes represent interquartile range for 40-gene bins, ranked by Euclidean distance to *POLR3G*. (**i**) GO enrichment analysis for top 400 genes (top 10 bins, highlighted in Figure 5e) with highest similarity to *POLR3G* regulatory profile. Individual genes encoding all RNA polymerase I, II, and III subunits are shown and highlighted as in 5c. (**j**) Gene set enrichment scores for 1662 transcription factor target gene sets ranked by pan-cancer *POLR3G* correlation scores. Scores for each individual factor ranked by normalized enrichment scores (NES) × −log_10_(FDR). Inset highlights CDKN1B and KDM5B separately for clarity. (**k**) Spearman’s rank correlation of GSEA scores with corresponding ChIP-seq signal at *POLR3G* promoter; factors with strongest regulatory prediction are highlighted. Color scheme same as in 5g. (**l**) Model: *POLR3G* expression and ultimately RPC7α availability is determined with a multitude of factors that either promote or restrict *POLR3G* gene activity. Biological replicates = 3; * *p* ≤ 0.05; ** *p* ≤ 0.01; *** *p* ≤ 0.001; **** *p* ≤ 0.0001.

Though our genomic survey reaffirms a central role for MYC in *POLR3G* regulation, MYC is known to regulate thousands of genes important for cell growth and more broadly recognized as a driver of transcriptional amplification rather than a factor responsible for selective gene activation [49,50]. This generality likely also applies to MXD4, given that MXD4 perturbs MYC-MAX activity, and KDM5B has similarly been linked to MYC-mediated transcription regulation [51]. An overlap analysis of individual MYC, MAX, MXD4, and KDM5B target gene sets reveals that a significant number of genes are targeted by all four factors (Figure 5a). Taken further, we find that the distribution of genes co-targeted by MYC, MAX, MXD4, and KDM5B have significantly higher POLR3G correlation scores in cancer, and these overlapping gene targets are enriched for factors that are similarly important for growth, including ribosome, spliceosome, and (other) RNA polymerase subunits (Figure 5b,c). A majority of genes encoding RNA polymerase I, II, and III subunits are, in fact, linked to all four regulatory factors, suggesting MYC, MAX, MXD4, and KDM5B broadly shape the expression and availability of most RNA polymerase subunits (Figure 5c). We confirm with RT−qPCR that overexpression of MYC indeed increases the expression of additional Pol III subunits, including *POLR3B*, *POLR3C*, and *POLR3F*, whereas *POLR3GL* mRNA levels are reduced (Figure 5d). This pattern contrasts that of MXD4 or KDM5B overexpression experiments, which together demonstrate that MXD4 and KDM5B functionally restrict the expression of genes encoding multiple Pol III subunits (Figure 5e,f). These results establish MXD4 and KDM5B as likely general determinants of transcription for multiple RNA polymerase subunits, potentially necessitating the multimodal regulatory platform observed for *POLR3G* expression. We therefore sought to further leverage global regulatory signatures to clarify features of particular significance for *POLR3G* expression.

### 3.11. Integrated Regulatory Signatures Implicate Additional Factors as Putative Determinants of POLR3G Expression in Cancer

The observed overlap of MYC-associated factors is indicative of regulatory similarity shared between *POLR3G* and genes encoding other Pol III subunits. To gain a better perspective on those features that are particularly relevant and potentially more specific to *POLR3G*, we expanded our analysis of target gene sets beyond MYC, MAX, MXD4, and KDM5B, to more than 1600 factors mapped with ChIP-atlas [24]. We determined the Euclidean distance between *POLR3G* and all other genes across the high-dimensional regulatory landscape, defined with the presence or absence of each factor. In this way, gene distances are a reflection of regulatory similarity and thus a useful method to identify genes with shared transcription factor paradigms (Figure 5g). Integration of gene-regulatory distances with our pan-cancer co-expression correlation results reveals a strong link between regulatory similarity and *POLR3G* correlation scores in cancer (Figure 5h). Subsetting on the top 400 genes, we find that genes with similar transcription factor profiles are enriched for ubiquitin ligase, mitochondrial, and spliceosomal proteins (Figure 5i). In this analysis, only *POLR3C*—a shared member of the RPC3−RPC6−RPC7 subcomplex—is included among the top set of genes with particularly strong regulatory similarity, suggesting a potentially unique subrepertoire of shared factors for *POLR3G* and *POLR3C* regulation (Figure 5i).

Complementary to the gene-distance analysis, distance-based methods applied to the presence or absence of genes within a TF regulatory set offer a top-down approach to assess similarity in TF target gene sets. Using this method, we find that TFs with strong ChIP-seq signal enrichment at genes with the highest *POLR3G* regulatory similarity tend to cluster, consistent with combinatorial transcription factor overlap across a multitude of target genes (Figure S13). Taken further, we profiled the enrichment of individual TF target gene sets among the ranked list of all pan-cancer *POLR3G* correlation scores. A gene set enrichment analysis (GSEA) specifically tallies enrichment scores based on the overrepresentation of a specific gene set. MYC-target genes, for example, are significantly enriched with respect to *POLR3G* correlation rankings, as expected for an MYC co-regulatory network (Figure 5j). This enrichment is similarly observed for MAX, MXD4, and KDM5B, consistent with the interplay of these factors with MYC and the repressive effects of MXD4 and KDM5B on *POLR3G* expression (Figure 5k). 

Integrating GSEA scores with the ChIP-seq signal observed for individual factors at the *POLR3G* promoter presents an additional indication of factors with particularly strong regulatory prediction. Both MAX and CDKN1B, which are highly enriched at the multi-promoter hub connecting *POLR3G* to neighboring genes (Figure 4e,f), are among the strongest features linked to *POLR3G* with this analysis (Figure 5l). ZNF687, a strong negative correlate identified in our co-expression correlation survey (Figure 1f) with signal enrichment at the *POLR3G* promoter (Figure 2a) is also among the strongest factors shared by *POLR3G* and related genes (Figure 5k). Altogether, these data implicate several additional factors, highlighting the overall complexity of combinatorial gene regulation and further supporting a multimodal model for *POLR3G* regulation underlying RPC7α re-emergence in cancer (Figure 5l).

## 4. Discussion

Our current study leverages large-scale multi-omic data to better understand the molecular underpinnings of *POLR3G* expression and, consequently, RPC7α upregulation in cancer. Through an integrative analysis of the genomic and epigenomic environment of *POLR3G* and in vitro functional experiments in multiple cell lines, our study reaffirms MYC as a central player in *POLR3G* expression while expanding on the list of players involved in dynamic *POLR3G* activity. Far from identifying a singular determinant of *POLR3G* expression, our results suggest that *POLR3G* mRNA levels are ultimately the product of multiple activating and repressive signals that converge on the *POLR3G* gene promoter, a gene-internal super-enhancer, and 3D chromatin architecture. We speculate that the multimodal nature of *POLR3G* regulation facilitates dynamic programming of RPC7α availability, as first proposed by Nouria Hernandez and colleagues [7]. 

Pluripotency factors OCT4 and NANOG, which play critical roles in *POLR3G* expression during early development, are notably omitted from the list of features that correlate with *POLR3G* levels in cancer, consistent with the absence of OCT4 and NANOG in most contexts (Figure S14). Instead, our study identifies Zinc finger proteins ZNF131 and ZNF207, which are more ubiquitously expressed across commonly used cell lines (Figure S15), among the multiple factors that promote *POLR3G* expression. ZNF131 was identified as the strongest positive TF correlate of *POLR3G* expression taken across all cancer subtypes, and we demonstrate that *POLR3G* expression is in fact sensitive to ZNF131 disruption and overexpression. Reports link ZNF131 to pluripotency maintenance and dynamic estrogen receptor-mediated regulation by functioning as a transcriptional co-activator or repressor [52,53]. ChIP data for ZNF131 are unfortunately limited, with only a single experimental replicate in HEK293 indicating some level of occupancy at the *POLR3G* locus. ZNF207, which has also been linked to pluripotency maintenance, undergoes isoform switching during development. We note that the isoform cloned and expressed in HEK293 (Figure S6) corresponds to ZNF207 isoform C (ENST00000394670.9), the dominant isoform expressed in hESCs thought to promote self-renewal and pluripotency in stem cells [33]. It is thus tempting to speculate that ZNF131 and ZNF207, which are more broadly expressed than OCT4 and NANOG, may functionally substitute for canonical pluripotency factors by potentially targeting similar gene-internal sequence elements residing within *POLR3G*. The gain of DNA methylation within this region in differentiated contexts, which may play an important role in preventing DNA binding by these and a multitude of other factors, remains a compelling regulatory mechanism for future investigation.

Analogous to the combination of features associated with enhanced *POLR3G* expression, our integrated survey implicates multiple factors in *POLR3G* silencing beyond DNA methylation of the gene-internal element. Antithetical to ZNF131, MXD4 was identified as the strongest negative TF correlate of *POLR3G* expression in cancer and, we show, is a negative determinant of *POLR3G* mRNA abundance. MXD4 opposes MYC by competing for heterodimerization with MAX, thereby intuitively countering the role of MYC in *POLR3G* regulation. However, we further show that the *POLR3G* promoter is targeted by KDM5B, a repressive lysine demethylase that is enriched for occupancy across a multi-promoter hub linking *POLR3G* to neighboring genes. KDM5B has been shown to play a direct role in MYC-mediated repression of *CDKN1A* [51], and we demonstrate that thousands of KDM5B target genes, enriched for genes central to growth, are shared by MYC, MAX, and MXD4. *POLR3G* expression is also inversely correlated with RARA and RXRA and rapidly sensitive to retinoic-acid-induced cellular differentiation. These and other factors identified at the *POLR3G* promoter together give rise to a multimodal model of *POLR3G* regulation, such that *POLR3G* expression is likely governed by the contextual balance of both positive and negative regulatory factors (Figure 5l). Understanding the multitude of factors that shape *POLR3G* expression may inform future strategies of inhibiting Pol III transcription and, particularly, the cancer-associated form of Pol III through inhibition of *POLR3G*. 

## 5. Conclusions

The regulatory underpinnings of *POLR3G* expression shape Pol III identity and downstream transcription. While multiple transcription factors have been implicated in *POLR3G* regulation, the intersection of these and potentially other factors has not been described. Through an integrative analysis of the *POLR3G* gene locus combined with functional experiments targeting specific factors, we show that *POLR3G* expression and ultimately RPC7α availability is determined with a multitude of factors that either promote or restrict *POLR3G* gene activity. These findings altogether uncover a multimodal platform for *POLR3G* expression, a feature that likely extends to other genes and which ostensibly connects multiple pathways to the dynamic regulation of Pol III transcription and related processes supporting cellular growth.

## Figures and Tables

**Table 1:** Primer sequences for molecular cloning.

Primer	Sequence
ZNF131-F	CGAGCTCGGATCCGCCACCATGGAGGCTGAAGAGACGATGG
ZNF131-R	CTTATCGTCGTCATCCTTGTAATCTTCTAAAACTGGCAGAGCTGTT
ZNF207-R	CTTATCGTCGTCATCCTTGTAATCGTAACGGCCACCTTGCGACATT
ZNF207-F	CGAGCTCGGATCCGCCACCATGGGTCGCAAGAAGAAGAAGCAG
pcDNA-BB-R	CATGGTGGCGGATCCGAGCT
pcDNA-BB-F	GATTACAAGGATGACGACGATAAGTGA

**Table 2:** siRNA sequences.

siRNA	Sequence	Concentration
ZNF207-siRNA1	rGrArUrGrArArArGrArCrGrArCrGrArCrUrUrCTT rGrArArGrUrCrGrUrCrGrUrCrUrUrUrCrArUrCTT	100 nM
ZNF207-siRNA2	rCrUrUrArGrCrUrArUrUrCrArUrUrGrCrArUrGTT rCrArUrGrCrArArUrGrArArUrArGrCrUrArArGTT	200 nM
ZNF131-siRNA1	rArArGrGrUrArUrUrGrArArArUrUrGrUrGrGrArArCTT rGrUrUrCrCrArCrArArUrUrUrCrArArUrArCrCrUrUTT	100 nM
ZNF131-siRNA2	rArArGrGrUrArCrUrGrArArGrUrArCrArUrGrUrArGTT rCrUrArCrArUrGrUrArCrUrUrCrArGrUrArCrCrUrUTT	100 nM
Scramble-siRNA	rUrUrCrUrCrCrGrArArCrGrUrGrUrCrArCrGrUTT rArCrGrUrGrArCrArCrGrUrUrCrGrGrArGrArATT	100 nM/200 nM

## Data Availability

The data presented in this study are available in this article (and Supplementary Materials), or otherwise publicly available through resources described in Section 2.8 Data Acquisition.

## Supplementary Materials

The following supporting information can be downloaded at: https://www.mdpi.com/article/10.3390/cancers15204995/s1. Figure S1: Additional antibody validation tests for cross-reactivity between reagents developed against RPC7a (POLR3G) and RPC7b (POLR3GL); Figure S2: Pan-cancer survival comparison of POLR3G and POLR3GL (RNA-seq; expression) and all other Pol III subunits (highlighted in gray) in relation to the full distribution of survival scores (data acquired from tcga-survival.com version 2.0 (Smith & Sheltzer, 2022)); Figure S3: A survey of POLR3G expression and RPC7a abundance across cell lines; Figure S4: Overexpression of MYC in HEK293T increases POLR3G expression and RPC7a abundance; Figure S5: MYC/MAX dimerization inhibitor 10058-F4 disrupts canonical MYC target genes in THP-1 monocytes, however alternate patterns are observed in HEK293T and A549. RT-qPCR analysis of canonical MYC target genes (CCND2 and CDK4) and genes negatively regulated by MYC (CDKN1A, CDKN1B) following exposure to 10058-F4 (150 uM, orange; 200 uM; blue), compared with DMSO control (gray); Figure S6: Overexpression of Zinc finger proteins ZNF131 and ZNF207 increase POLR3G mRNA abundance in HEK293T cells. Figure S7: Pan-cancer survival comparison of POLR3G to POLR3GL (DNA methylation) and all other Pol III subunits (highlighted in gray) in relation to the full distribution of survival scores (data acquired from tcga-survival.com version 2.0 (Smith & Sheltzer, 2022)). Figure S8: Downregulation of POLR3G expression in response to retinoic acid (RA) in NTERA-2 cells and THP-1 monocytes. Figure S9: Overexpression of MXD4 and KDM5B in HEK293T cells. Figure S10: Pan-cancer survival comparison of MXD4 to all basic helix-loop-helix (bHLH)-containing trans-cription factors (RNA-seq; expression) in relation to the full distribution of survival scores (data acquired from tcga-survival.com version 2.0 (Smith & Sheltzer, 2022)). Figure S11: xamination of the strongest negative chromatin correlates with POLR3G expression in cancer (top); Figure S12: POLR3G neighboring genes, CETN3, MBLAC2, LYSMD3, and ADGRV1, correlate with POLR3G expression in cancer. Figure S13: Visualization of transcription factor gene set similarity, as defined by the presence or absence at the promoter (+/− 1 Kb) of 19,022 target genes; Figure S14: Expression of Pluripotency factors OCT4 and NANOG is largely restricted to a limited number of cell lines; Figure S15: Expression of pluripotency-related Zinc finger proteins ZNF131 and ZNF207 is generally ubiquitous across most cell lines.

## References

[1] Yeganeh M., Hernandez N. (2020). RNA polymerase III transcription as a disease factor. Genes Dev..

[2] Willis I.M., Moir R.D. (2018). Signaling to and from the RNA Polymerase III Transcription and Processing Machinery. Annu. Rev. Biochem..

[3] Vannini A., Cramer P. (2012). Conservation between the RNA polymerase I, II, and III transcription initiation machineries. Mol. Cell.

[4] Wang Z., Roeder R.G. (1997). Three human RNA polymerase III-specific subunits form a subcomplex with a selective function in specific transcription initiation. Genes Dev..

[5] Kenneth N.S., Marshall L., White R.J. (2008). Recruitment of RNA polymerase III in vivo. Nucleic Acids Res..

[6] Wang Q., Li S., Wan F., Xu Y., Wu Z., Cao M., Lan P., Lei M., Wu J. (2021). Structural insights into transcriptional regulation of human RNA polymerase III. Nat. Struct. Mol. Biol..

[7] Renaud M., Praz V., Vieu E., Florens L., Washburn M.P., l’Hôte P., Hernandez N. (2014). Gene duplication and neofunctionalization: POLR3G and POLR3GL. Genome Res..

[8] Schwartz L.B., Sklar V.E., Jaehning J.A., Weinmann R., Roeder R.G. (1974). Isolation and partial characterization of the multiple forms of deoxyribonucleic acid-dependent ribonucleic acid polymerase in the mouse myeloma, MOPC 315. J. Biol. Chem..

[9] Haurie V., Durrieu-Gaillard S., Dumay-Odelot H., Da Silva D., Rey C., Prochazkova M., Roeder R.G., Besser D., Teichmann M. (2010). Two isoforms of human RNA polymerase III with specific functions in cell growth and transformation. Proc. Natl. Acad. Sci. USA.

[10] Petrie J.L., Swan C., Ingram R.M., Frame F.M., Collins A.T., Dumay-Odelot H., Teichmann M., Maitland N.J., White R.J. (2019). Effects on prostate cancer cells of targeting RNA polymerase III. Nucleic Acids Res..

[11] McQueen C., Hughes G.L., Pownall M.E. (2019). Skeletal muscle differentiation drives a dramatic downregulation of RNA polymerase III activity and differential expression of Polr3g isoforms. Dev. Biol..

[12] Van Bortle K., Marciano D.P., Liu Q., Chou T., Lipchik A.M., Gollapudi S., Geller B.S., Monte E., Kamakaka R.T., Snyder M.P. (2022). A cancer-associated RNA polymerase III identity drives robust transcription and expression of snaR-A noncoding RNA. Nat. Commun..

[13] Lautré W., Richard E., Feugeas J.-P., Dumay-Odelot H., Teichmann M. (2022). The POLR3G Subunit of Human RNA Polymerase III Regulates Tumorigenesis and Metastasis in Triple-Negative Breast Cancer. Cancers.

[14] Liu X., Zhang W., Wang H., Lai C.H., Xu K., Hu H. (2020). Increased expression of POLR3G predicts poor prognosis in transitional cell carcinoma. PeerJ.

[15] Yang J., Wang F., Zhong S., Chen B. (2021). Identification of hub genes with prognostic values in multiple myeloma by bioinformatics analysis. Hematology.

[16] Dai X., Jiang W., Ma L., Sun J., Yan X., Qian J., Wang Y., Shi Y., Ni S., Yao N. (2021). A metabolism-related gene signature for predicting the prognosis and therapeutic responses in patients with hepatocellular carcinoma. Ann. Transl. Med..

[17] Sun N., Chu J., Hu W., Chen X., Yi N., Shen Y. (2022). A novel 14-gene signature for overall survival in lung adenocarcinoma based on the Bayesian hierarchical Cox proportional hazards model. Sci. Rep..

[18] Wong R.C., Pollan S., Fong H., Ibrahim A., Smith E.L., Ho M., Laslett A.L., Donovan P.J. (2011). A novel role for an RNA polymerase III subunit POLR3G in regulating pluripotency in human embryonic stem cells. Stem Cells.

[19] Smith J.C., Sheltzer J.M. (2022). Genome-wide identification and analysis of prognostic features in human cancers. Cell Rep..

[20] Hutter C., Zenklusen J.C. (2018). The Cancer Genome Atlas: Creating Lasting Value beyond Its Data. Cell.

[21] Corces M.R., Granja J.M., Shams S., Louie B.H., Seoane J.A., Zhou W., Silva T.C., Groeneveld C., Wong C.K., Cho S.W. (2018). The chromatin accessibility landscape of primary human cancers. Science.

[22] Ochsner S.A., Abraham D., Martin K., Ding W., McOwiti A., Kankanamge W., Wang Z., Andreano K., Hamilton R.A., Chen Y. (2019). The Signaling Pathways Project, an integrated ’omics knowledgebase for mammalian cellular signaling pathways. Sci. Data.

[23] Uhlén M., Fagerberg L., Hallström B.M., Lindskog C., Oksvold P., Mardinoglu A., Sivertsson Å., Kampf C., Sjöstedt E., Asplund A. (2015). Proteomics. Tissue-based map of the human proteome. Science.

[24] Zou Z., Ohta T., Miura F., Oki S. (2022). ChIP-Atlas 2021 update: A data-mining suite for exploring epigenomic landscapes by fully integrating ChIP-seq, ATAC-seq and Bisulfite-seq data. Nucleic Acids Res..

[25] Consortium E.P. (2012). An integrated encyclopedia of DNA elements in the human genome. Nature.

[26] Wang Y., Song C., Zhao J., Zhang Y., Zhao X., Feng C., Zhang G., Zhu J., Wang F., Qian F. (2023). SEdb 2.0: A comprehensive super-enhancer database of human and mouse. Nucleic Acids Res..

[27] Wu T., Hu E., Xu S., Chen M., Guo P., Dai Z., Feng T., Zhou L., Tang W., Zhan L. (2021). clusterProfiler 4.0: A universal enrichment tool for interpreting omics data. Innovation.

[28] Girbig M., Misiaszek A.D., Vorländer M.K., Lafita A., Grötsch H., Baudin F., Bateman A., Müller C.W. (2021). Cryo-EM structures of human RNA polymerase III in its unbound and transcribing states. Nat. Struct. Mol. Biol..

[29] Ramsay E.P., Abascal-Palacios G., Daiß J.L., King H., Gouge J., Pilsl M., Beuron F., Morris E., Gunkel P., Engel C. (2020). Structure of human RNA polymerase III. Nat. Commun..

[30] Li L., Yu Z., Zhao D., Ren Y., Hou H., Xu Y. (2021). Structure of human RNA polymerase III elongation complex. Cell Res..

[31] Cheng R., Van Bortle K. (2022). RNA polymerase III transcription and cancer: A tale of two RPC7 subunits. Front. Mol. Biosci..

[32] Bailey T.L. (2021). STREME: Accurate and versatile sequence motif discovery. Bioinformatics.

[33] Fang F., Xia N., Angulo B., Carey J., Cady Z., Durruthy-Durruthy J., Bennett T., Sebastiano V., Reijo Pera R.A. (2018). A distinct isoform of ZNF207 controls self-renewal and pluripotency of human embryonic stem cells. Nat. Commun..

[34] Chaudhary J., Skinner M.K. (1999). Basic helix-loop-helix proteins can act at the E-box within the serum response element of the c-fos promoter to influence hormone-induced promoter activation in Sertoli cells. Mol. Endocrinol..

[35] Wang R., Xu Q., Wang C., Tian K., Wang H., Ji X. (2023). Multiomic analysis of cohesin reveals that ZBTB transcription factors contribute to chromatin interactions. Nucleic Acids Res..

[36] Huang M.J., Cheng Y.C., Liu C.R., Lin S., Liu H.E. (2006). A small-molecule c-Myc inhibitor, 10058-F4, induces cell-cycle arrest, apoptosis, and myeloid differentiation of human acute myeloid leukemia. Exp. Hematol..

[37] García-Gutiérrez L., Delgado M.D., León J. (2019). MYC Oncogene Contributions to Release of Cell Cycle Brakes. Genes.

[38] Iguchi T., Aoki K., Ikawa T., Taoka M., Taya C., Yoshitani H., Toma-Hirano M., Koiwai O., Isobe T., Kawamoto H. (2015). BTB-ZF Protein Znf131 Regulates Cell Growth of Developing and Mature T Cells. J. Immunol..

[39] Bastien J., Rochette-Egly C. (2004). Nuclear retinoid receptors and the transcription of retinoid-target genes. Gene.

[40] Chandra V., Wu D., Li S., Potluri N., Kim Y., Rastinejad F. (2017). The quaternary architecture of RARβ-RXRα heterodimer facilitates domain-domain signal transmission. Nat. Commun..

[41] Chatagnon A., Veber P., Morin V., Bedo J., Triqueneaux G., Sémon M., Laudet V., d’Alché-Buc F., Benoit G. (2015). RAR/RXR binding dynamics distinguish pluripotency from differentiation associated cis-regulatory elements. Nucleic Acids Res..

[42] Lund R.J., Rahkonen N., Malonzo M., Kauko L., Emani M.R., Kivinen V., Närvä E., Kemppainen E., Laiho A., Skottman H. (2017). RNA Polymerase III Subunit POLR3G Regulates Specific Subsets of PolyA. Stem Cell Rep..

[43] Heim K.C., White K.A., Deng D., Tomlinson C.R., Moore J.H., Freemantle S.J., Spinella M.J. (2007). Selective repression of retinoic acid target genes by RIP140 during induced tumor cell differentiation of pluripotent human embryonal carcinoma cells. Mol. Cancer.

[44] Dimberg A., Bahram F., Karlberg I., Larsson L.G., Nilsson K., Oberg F. (2002). Retinoic acid-induced cell cycle arrest of human myeloid cell lines is associated with sequential down-regulation of c-Myc and cyclin E and posttranscriptional up-regulation of p27(Kip1). Blood.

[45] Hurlin P.J., Quéva C., Koskinen P.J., Steingrímsson E., Ayer D.E., Copeland N.G., Jenkins N.A., Eisenman R.N. (1995). Mad3 and Mad4: Novel Max-interacting transcriptional repressors that suppress c-myc dependent transformation and are expressed during neural and epidermal differentiation. EMBO J..

[46] Ostrowski J., Kuhns J.E., Lupisella J.A., Manfredi M.C., Beehler B.C., Krystek S.R., Bi Y., Sun C., Seethala R., Golla R. (2007). Pharmacological and x-ray structural characterization of a novel selective androgen receptor modulator: Potent hyperanabolic stimulation of skeletal muscle with hypostimulation of prostate in rats. Endocrinology.

[47] Wang H., Li B., Zuo L., Wang B., Yan Y., Tian K., Zhou R., Wang C., Chen X., Jiang Y. (2022). The transcriptional coactivator RUVBL2 regulates Pol II clustering with diverse transcription factors. Nat. Commun..

[48] Xhabija B., Kidder B.L. (2019). KDM5B is a master regulator of the H3K4-methylome in stem cells, development and cancer. Semin. Cancer Biol..

[49] Stine Z.E., Walton Z.E., Altman B.J., Hsieh A.L., Dang C.V. (2015). MYC, Metabolism, and Cancer. Cancer Discov..

[50] Nie Z., Hu G., Wei G., Cui K., Yamane A., Resch W., Wang R., Green D.R., Tessarollo L., Casellas R. (2012). c-Myc is a universal amplifier of expressed genes in lymphocytes and embryonic stem cells. Cell.

[51] Wong P.P., Miranda F., Chan K.V., Berlato C., Hurst H.C., Scibetta A.G. (2012). Histone demethylase KDM5B collaborates with TFAP2C and Myc to repress the cell cycle inhibitor p21(cip) (CDKN1A). Mol. Cell Biol..

[52] Garipler G., Lu C., Morrissey A., Lopez-Zepeda L.S., Pei Y., Vidal S.E., Zen Petisco Fiore A.P., Aydin B., Stadtfeld M., Ohler U. (2022). The BTB transcription factors ZBTB11 and ZFP131 maintain pluripotency by repressing pro-differentiation genes. Cell Rep..

[53] Han X., Guo J., Deng W., Zhang C., Du P., Shi T., Ma D. (2008). High-throughput cell-based screening reveals a role for ZNF131 as a repressor of ERalpha signaling. BMC Genom..

