# Supplementary material for: A Combinatorial Regulatory Platform Determines Expression of RNA Polymerase III Subunit RPC7α (POLR3G) in Cancer
Source: Cancers (Basel). 2023 Oct 15;15(20):4995. doi: 10.3390/cancers15204995 (PMC10605170; doi:10.3390/cancers15204995)

Uncropped WB

Cancers-2070777

# Corresponding to Fig1.b

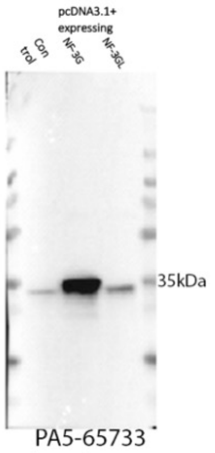

PA5-65733
L58-R104

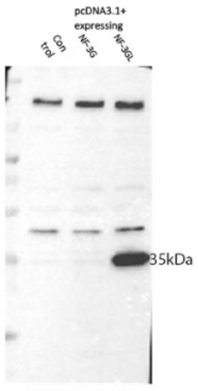

NBP1-79826
P166-E215

Corresponding to Fig2.f

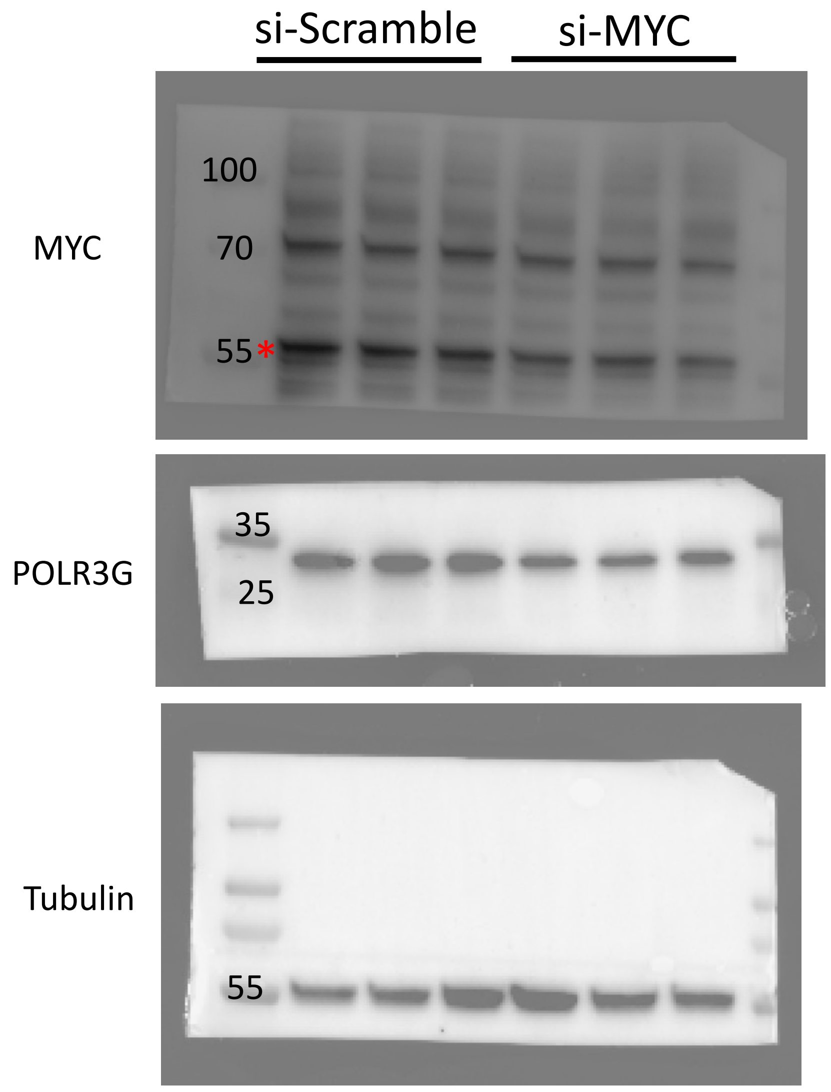

Corresponding to Fig2. l

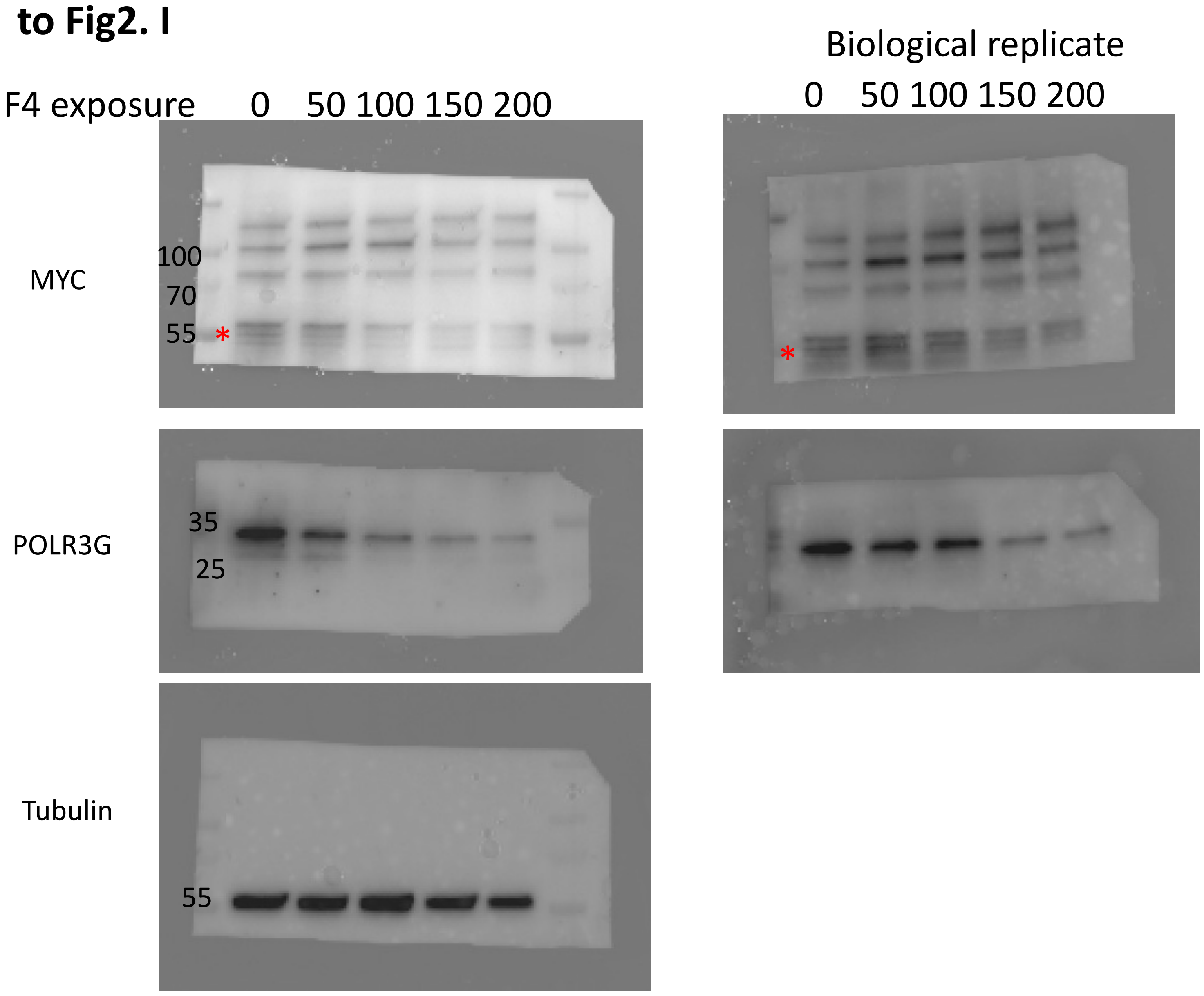

Corresponding to Fig2.i

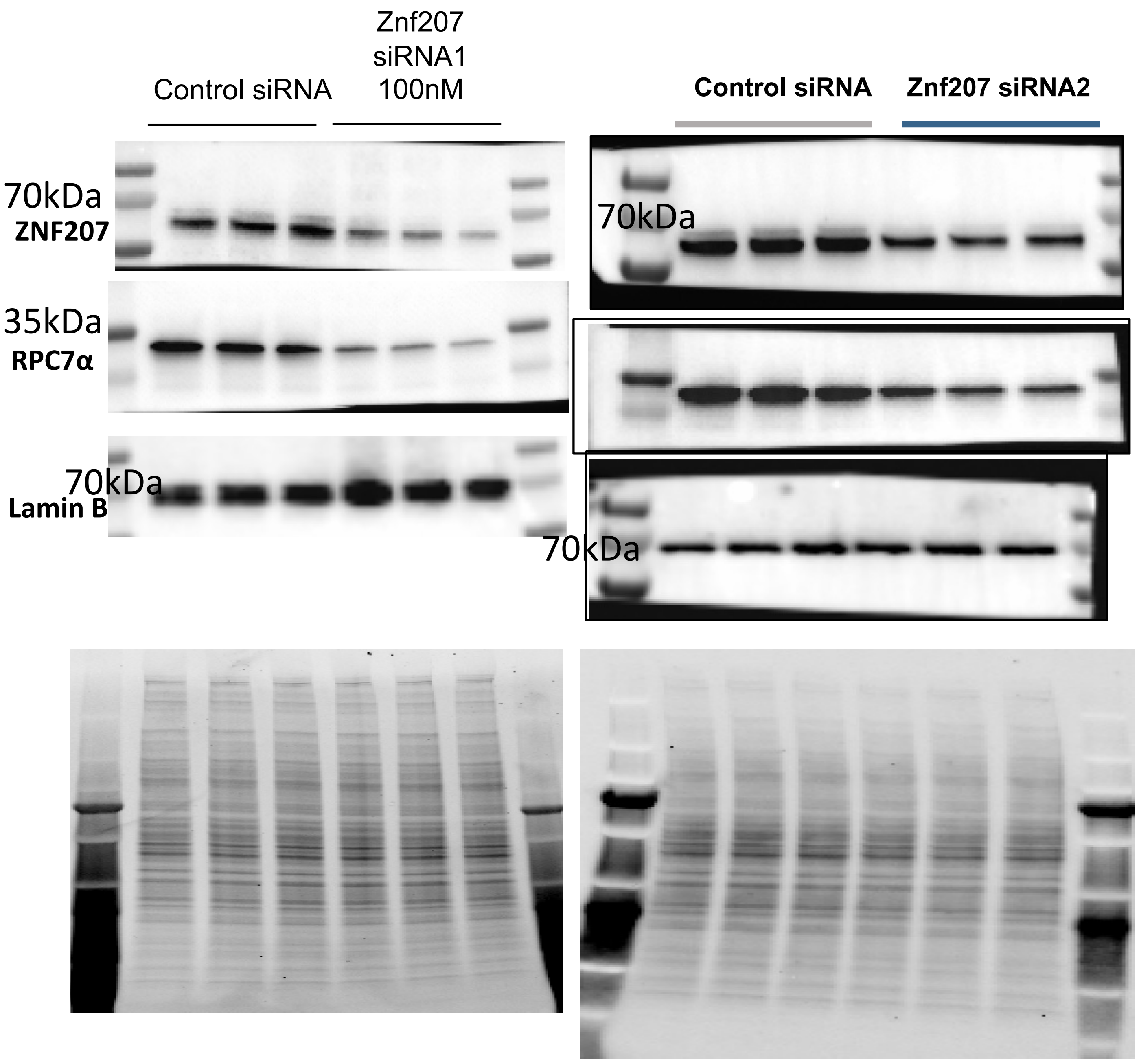

Corresponding to Fig2.o

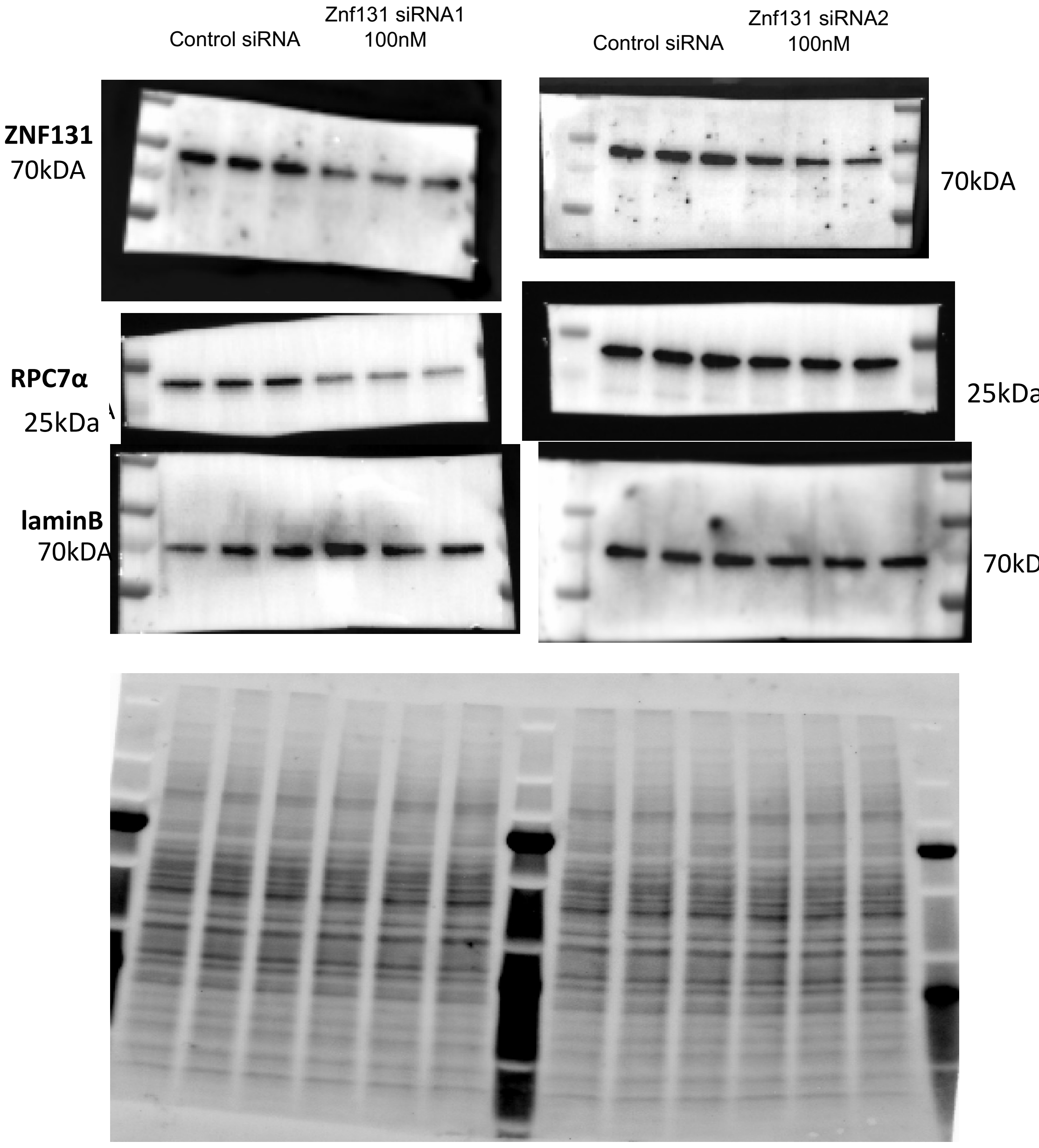

Corresponding to Fig3.g

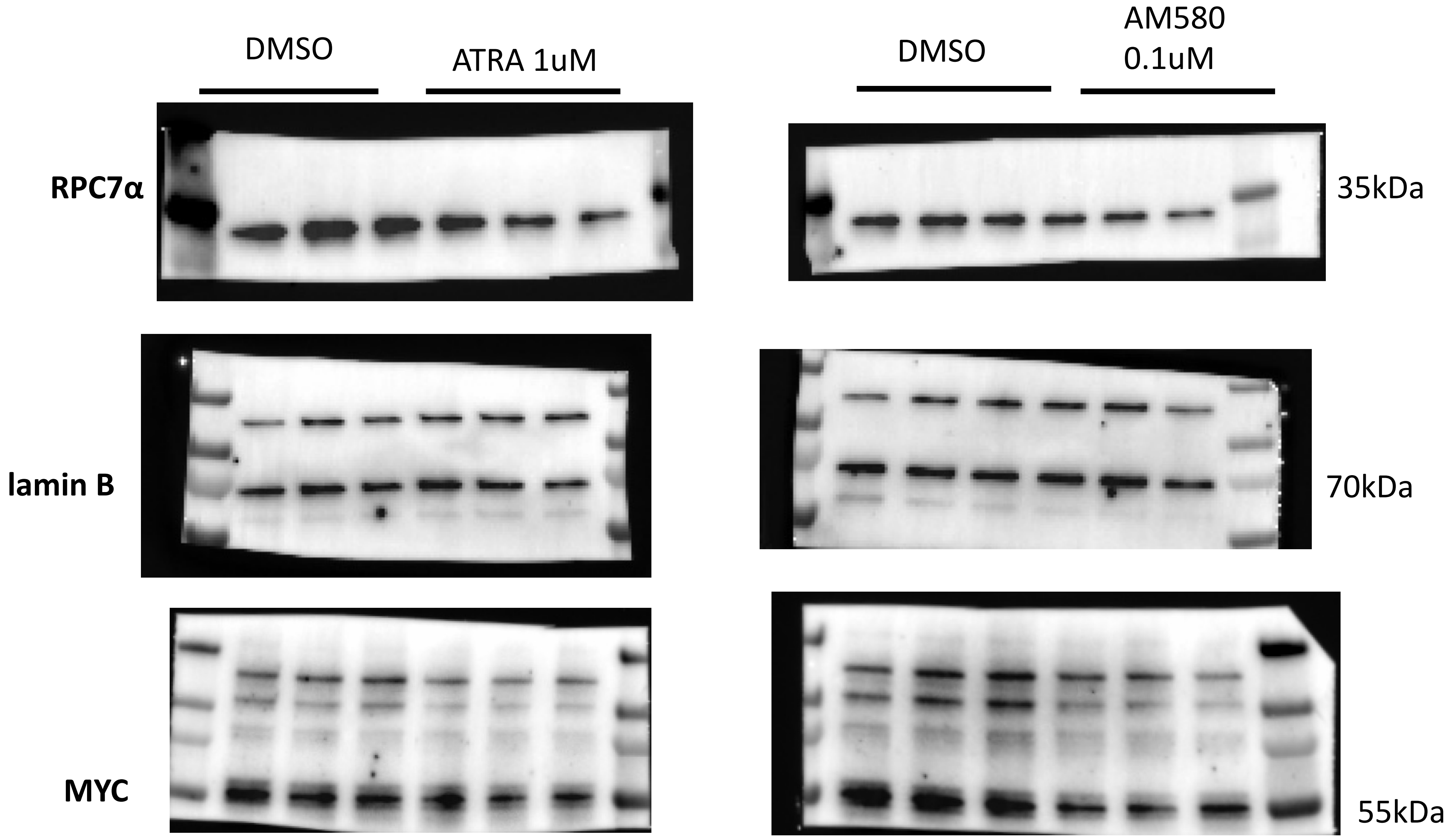

Corresponding **to** Supplemental figure 1

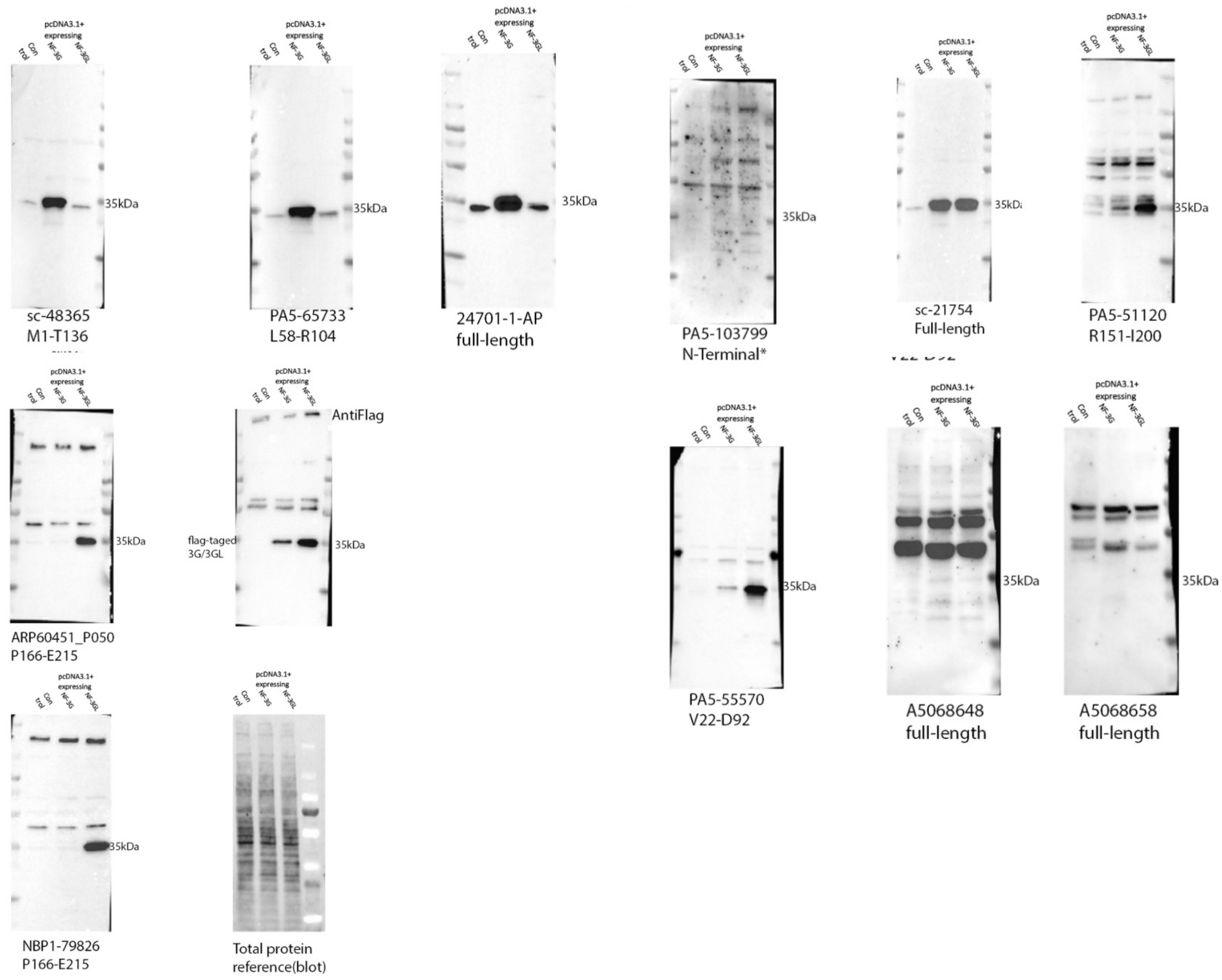

Corresponding to Supplemental figure 3.d

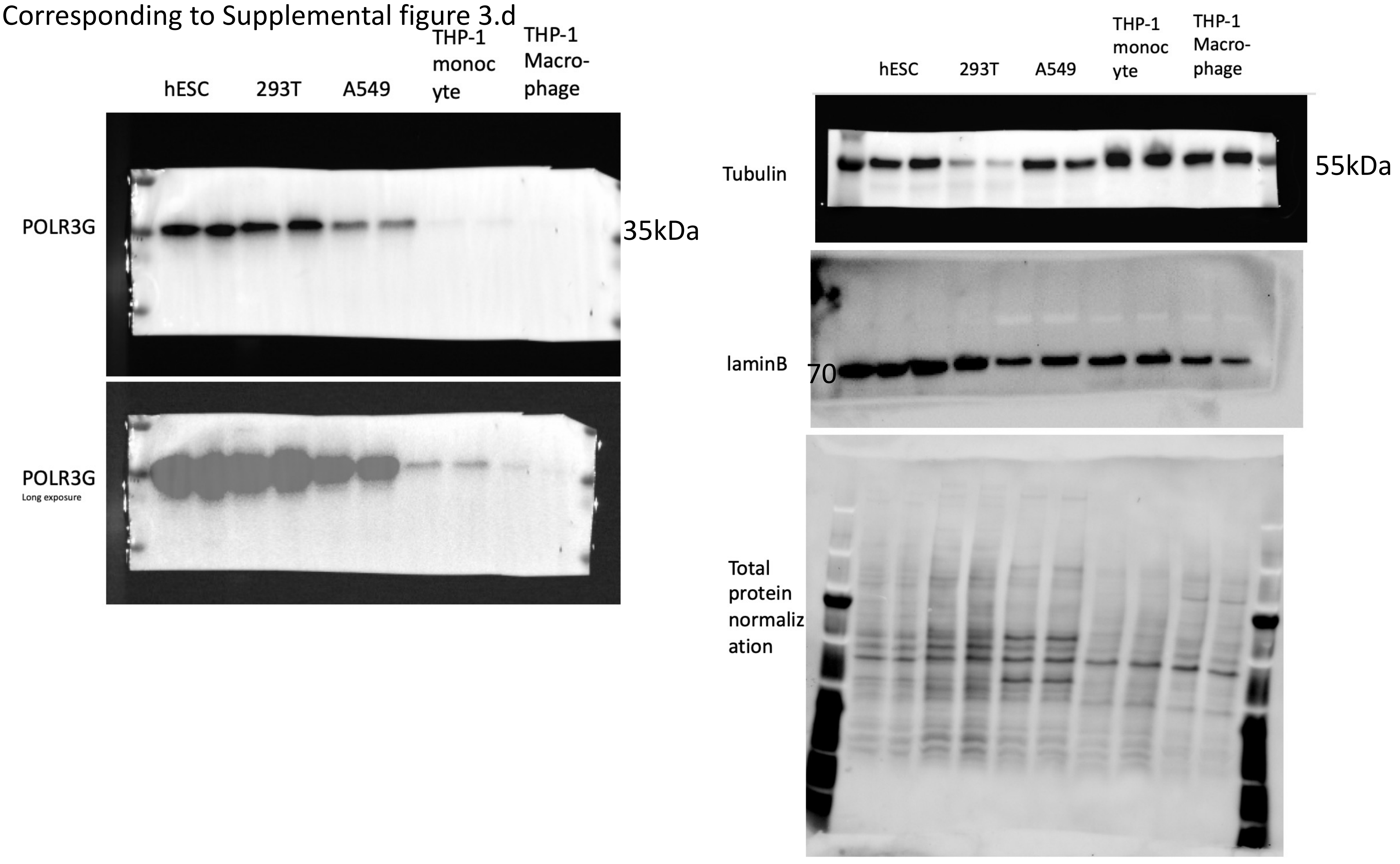

Corresponding to Supplemental figure 4 c

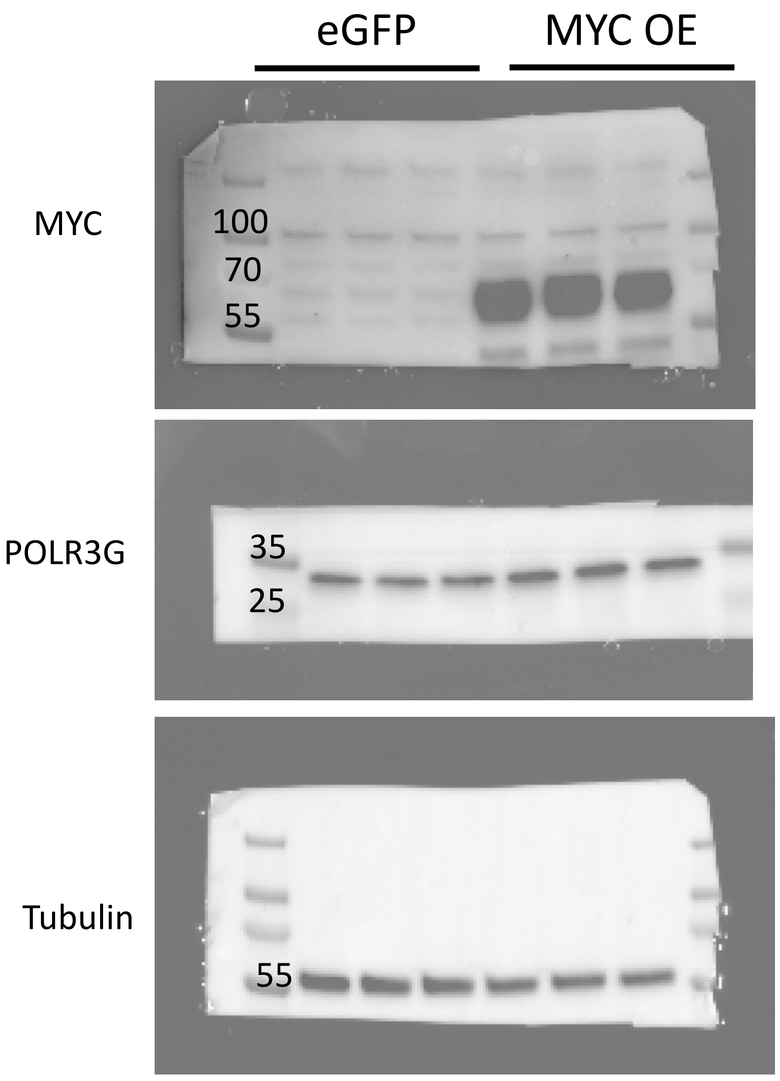

Corresponding to Supplemental figure 6

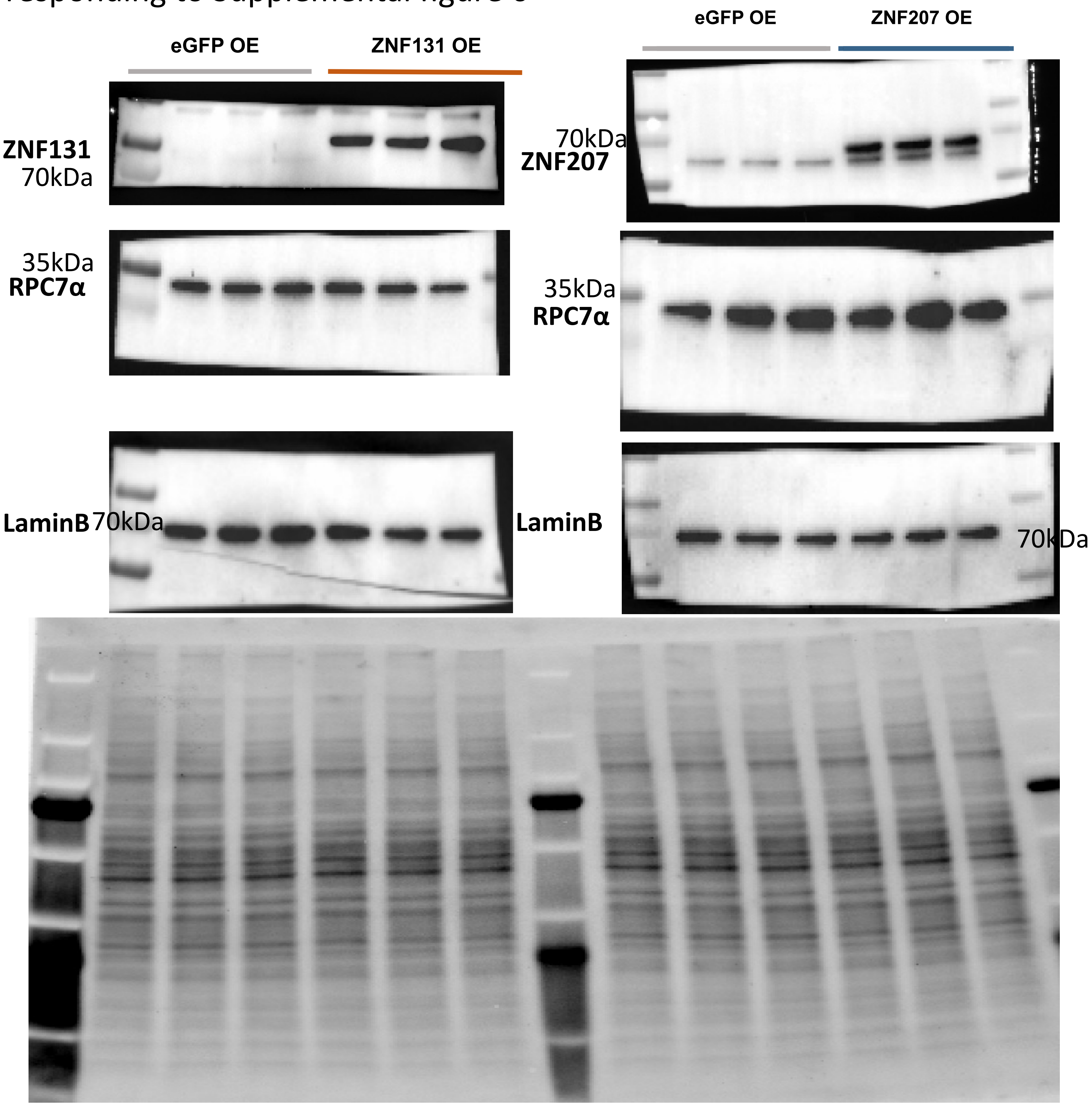

Corresponding to Supplemental figure 9

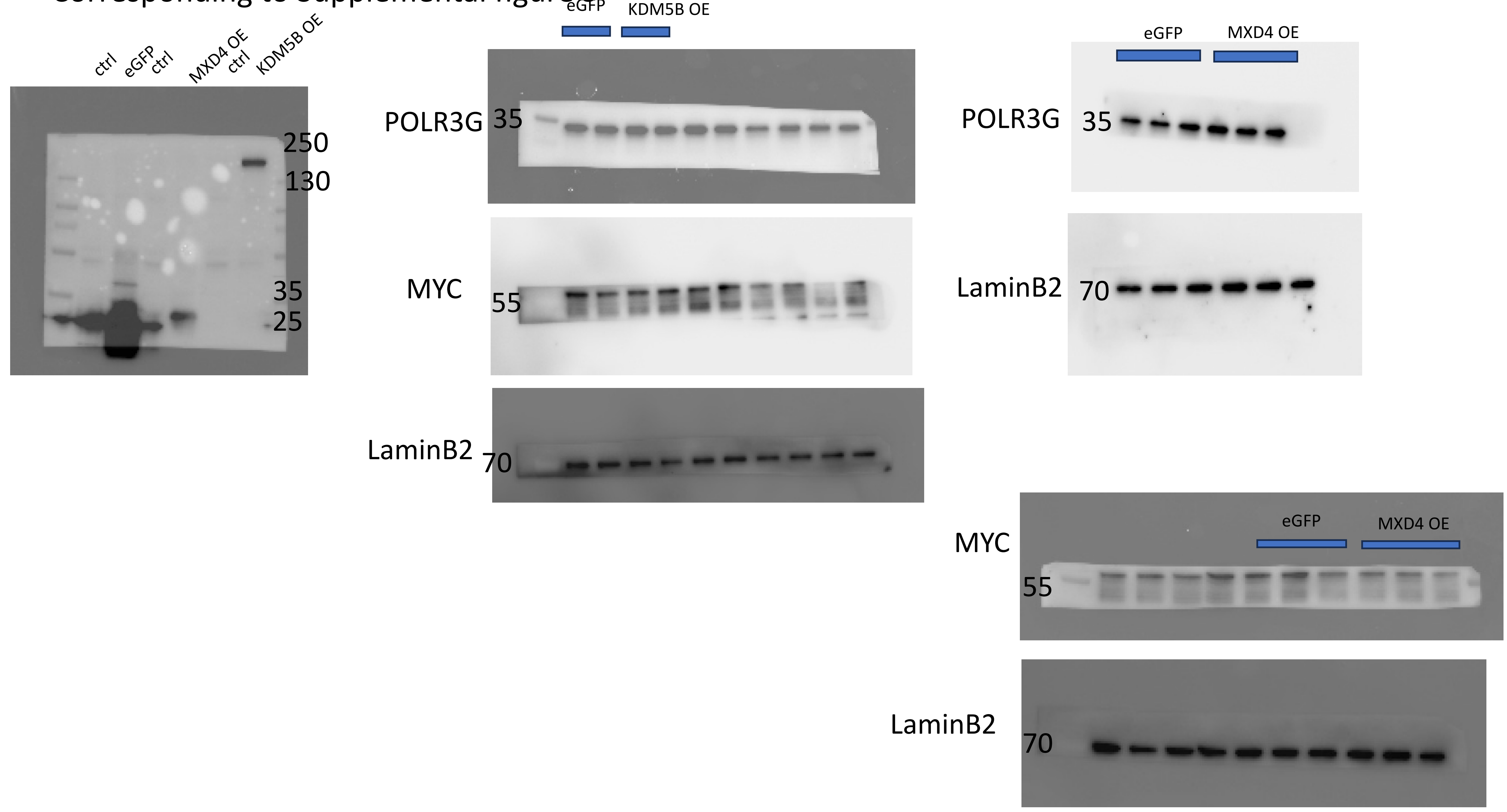

Supplement: Supplementary file 1 [file cancers-15-04995-s001.zip › cancers-2645739-original-images.pdf]
