# Supplementary material for: A Combinatorial Regulatory Platform Determines Expression of RNA Polymerase III Subunit RPC7α (POLR3G) in Cancer
Source: Cancers (Basel). 2023 Oct 15;15(20):4995. doi: 10.3390/cancers15204995 (PMC10605170; doi:10.3390/cancers15204995)
Supplement: Supplementary file 1 [file cancers-15-04995-s001.zip › cancers-2645739-supplementary.pdf]

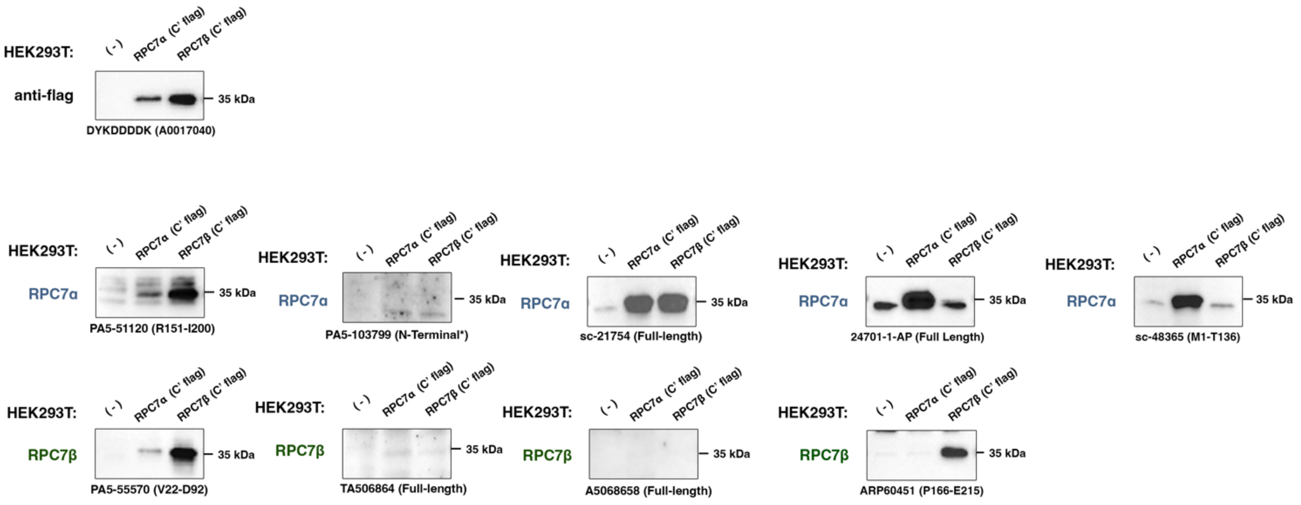

Supplemental Figure S1. Additional antibody validation tests for cross-reactivity between reagents developed against RPC7α (POLR3G) and RPC7β (POLR3GL). Immunoblot analysis of commercial antibody specificity for RPC7α (POLR3G) and RPC7β (POLR3GL) in HEK293T cell lysates with ectopic POLR3G or POLR3GL (pcDNA3.1) overexpression. (-) non-transfected HEK293T cells. Cross-reactive antibodies detect both RPC7α (POLR3G) and RPC7β (POLR3GL), whereas non-reactive antibodies do not detect either RPC7α (POLR3G) and RPC7β (POLR3GL).

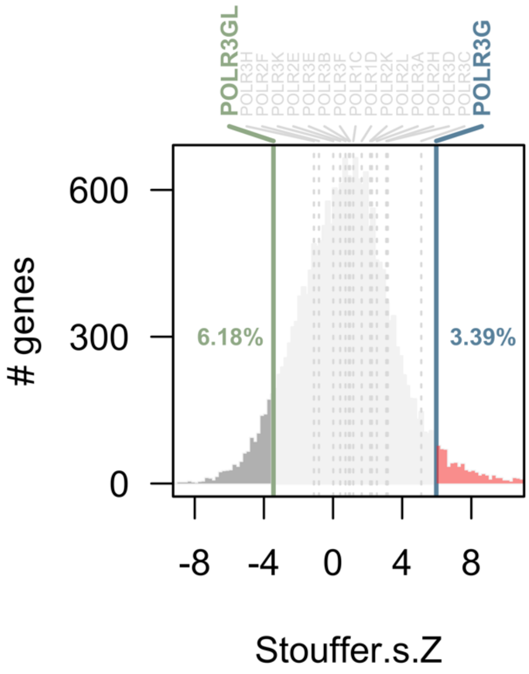

Supplemental Figure S2. Pan-cancer survival comparison of *POLR3G* and *POLR3GL* (**RNA-seq; expression**) and all other Pol III subunits (highlighted in gray) in relation to the full distribution of survival scores (data acquired from tcga-survival.com version 2.0 (Smith & Sheltzer, 2022)). Percent of genes with higher (POLR3G, blue) or lower (POLR3GL, green) pan-cancer z-scores are highlighted.

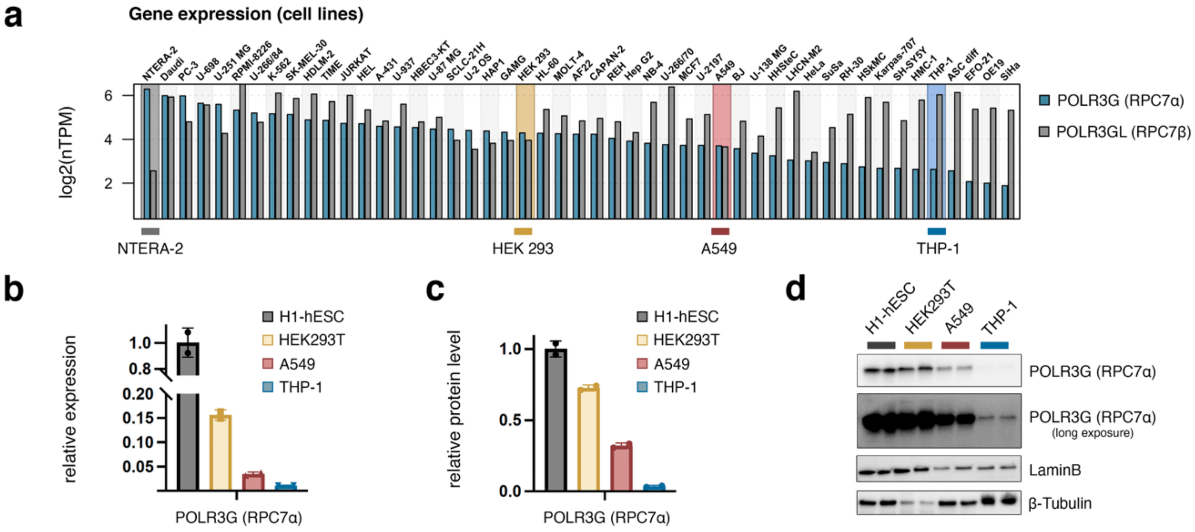

Supplemental Figure S3. A survey of *POLR3G* expression and RPC7α abundance across cell lines. (**a**) POLR3G and POLR3GL gene expression profiles across ~50 human cell lines (nTPM = normalized transcript per million; proteinatlas.org). Highlighted cell lines (NTERA-2, HEK293, A549, THP-1) relevant to cell lines in following *in vitro* experiments. (**b**) RT-qPCR analysis of POLR3G mRNA levels in H1-hESC, HEK293T, A549, and THP-1 cells. (**c, d**) Immunoblots and quantification of RPC7α (POLR3G), LaminB, and β-tubulin protein levels in H1-hESC, HEK293T, A549, and THP-1 cells. Protein abundances were calculated by normalizing to the average intensity of reference protein Lamin B2, β-tubulin and total protein.

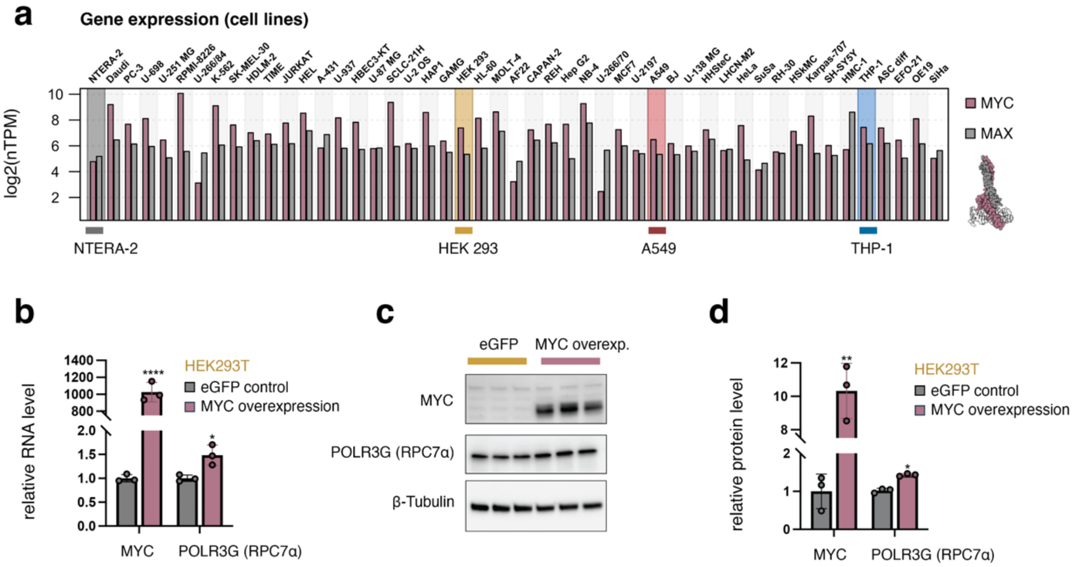

Supplemental Figure S4. Overexpression of MYC in HEK293T increases *POLR3G* expression and RPC7α abundance. (**a**) MYC and MAX gene expression profiles across ~50 human cell lines (nTPM = normalized transcript per million; proteinatlas.org). (**b**) RT-qPCR analysis of MYC and POLR3G mRNA levels in HEK293T following overexpression of MYC (pcDNA3.1) compared to GFP (pcDNA3.1; control). (**c, d**) Immunoblots and quantification of MYC and RPC7α (POLR3G) protein levels in HEK293T following overexpression of MYC (pcDNA3.1) compared to GFP (pcDNA3.1; control). $^{*}P \leq 0.05$; $^{**}P \leq 0.01$; $^{****}P \leq 0.0001$.

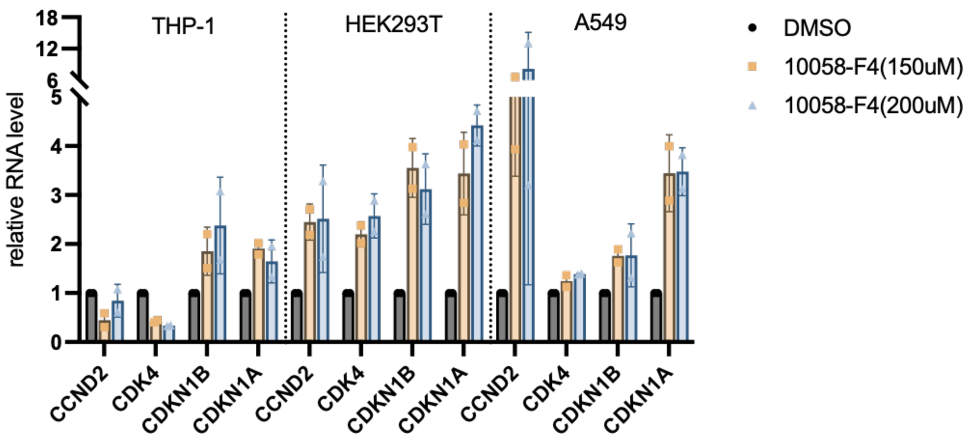

Supplemental Figure S5. MYC/MAX dimerization inhibitor 10058-F4 disrupts canonical MYC target genes in THP-1 monocytes, however alternate patterns are observed in HEK293T and A549. RT-qPCR analysis of canonical MYC target genes (CCND2 and CDK4) and genes negatively regulated by MYC (CDKN1A, CDKN1B) following exposure to 10058-F4 (150uM, orange; 200 uM; blue), compared with DMSO control (gray). CCND2 and CDK4 are downregulated following MYC/MAX dimerization in THP-1; this canonical effect is not observed in HEK293T and A549 cell lines.

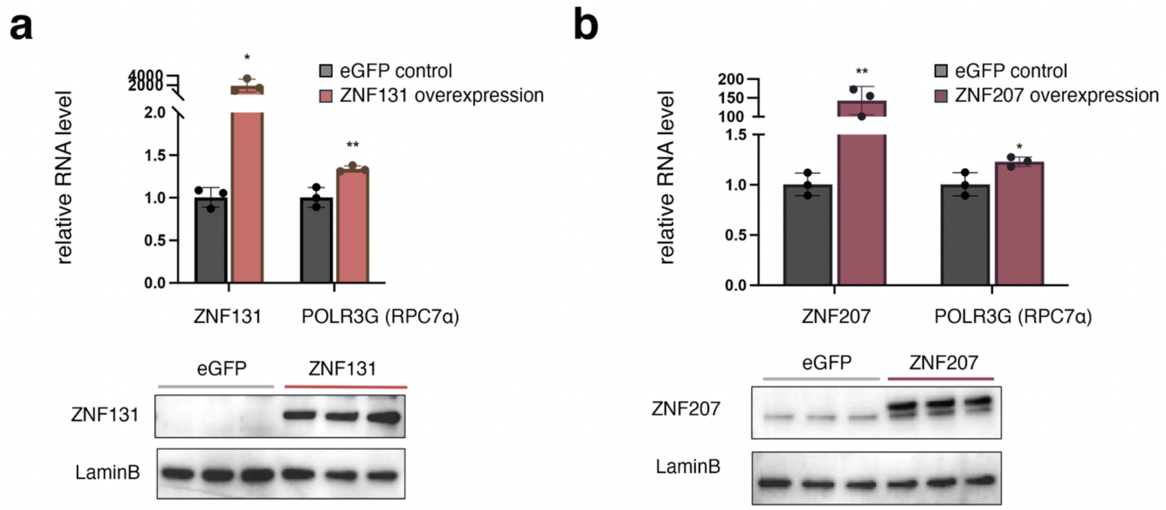

Supplemental Figure S6. Overexpression of Zinc finger proteins ZNF131 and ZNF207 increase *POLR3G* mRNA abundance in HEK293T cells. (**a**) RT-qPCR analysis of ZNF131 and POLR3G mRNA abundance in HEK293T cells overexpressing ZNF131 (pcDNA3.1) compared with eGFP (pcDNA3.1; control). Bottom: immunoblot validation of ZNF131 overexpression. (**b**) RT-qPCR analysis of ZNF207 and POLR3G mRNA abundance in HEK293T cells overexpressing ZNF207 (pcDNA3.1) compared with eGFP (pcDNA3.1; control). Bottom: immunoblot validation of ZNF207 overexpression. *$P \leq 0.05$; **$P \leq 0.01$.

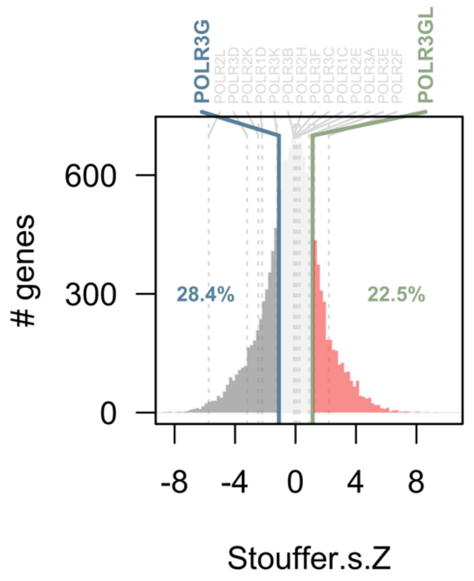

Supplemental Figure S 7. Pan-cancer survival comparison of *POLR3G* to *POLR3GL* (**DNA methylation**) and all other Pol III subunits (highlighted in gray) in relation to the full distribution of survival scores (data acquired from tcga-survival.com version 2.0 (Smith & Sheltzer, 2022)). Percent of genes with higher (POLR3GL, green) or lower (POLR3G, blue) pan-cancer z-scores are highlighted.

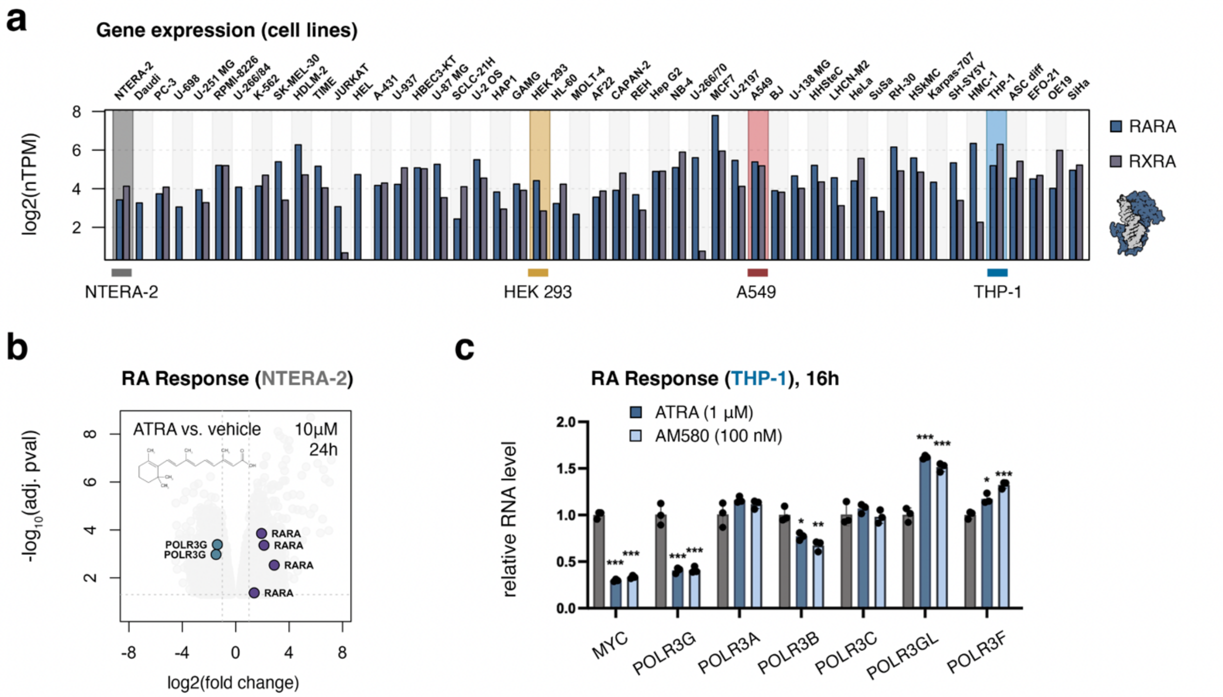

Supplemental Figure S8. Downregulation of *POLR3G* expression in response to retinoic acid (RA) in NTERA-2 cells and THP-1 monocytes. (**a**) RARA and RXRA gene expression profiles across ~50 human cell lines (nTPM = normalized transcript per million; proteinatlas.org). (**b**) Volcano plot analysis of differential gene expression in NTERA-2 cells following 24h ATRA exposure (data from pubmed ID: 17880687); multiple points correspond to unique array gene probes). (**c**) RT-qPCR analysis of MYC, POLR3G, POLR3A, POLR3B, POLR3C, POLR3GL, and POLR3F mRNA abundance in THP-1 monocytes following 16-hour treatment with ATRA (dark blue) or AM580 (light blue), an RARA selective agonist. *$P \leq 0.05$; **$P \leq 0.01$; ***$P \leq 0.001$.

Supplemental Figure S9. Overexpression of MXD4 and KDM5B in HEK293T cells. Immunoblot validation of MXD4 and KDM5B overexpression in HEK293T cells. Ctrl = non-transfected cells, transfection of flag-tagged eGFP (27 kDa), MXD4 (23.5 kDa), and KDM5B (175 kDa) pcDNA3.1 were confirmed with antibodies against DYKDDDK (A0017040).

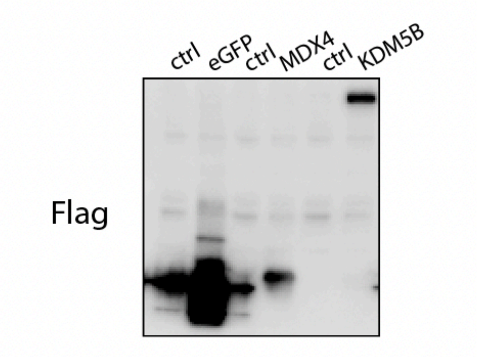

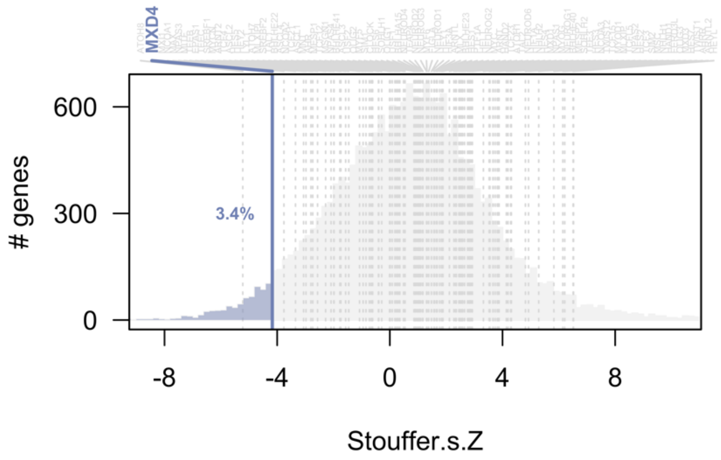

Supplemental Figure S 10. Pan-cancer survival comparison of MXD4 to all basic helix-loop-helix (bHLH)-containing trans-cription factors (**RNA-seq; expression**) in relation to the full distribution of survival scores (data acquired from tcga-survival.com version 2.0 (Smith & Sheltzer, 2022)). Percent of genes with lower pan-cancer survival scores are highlighted.

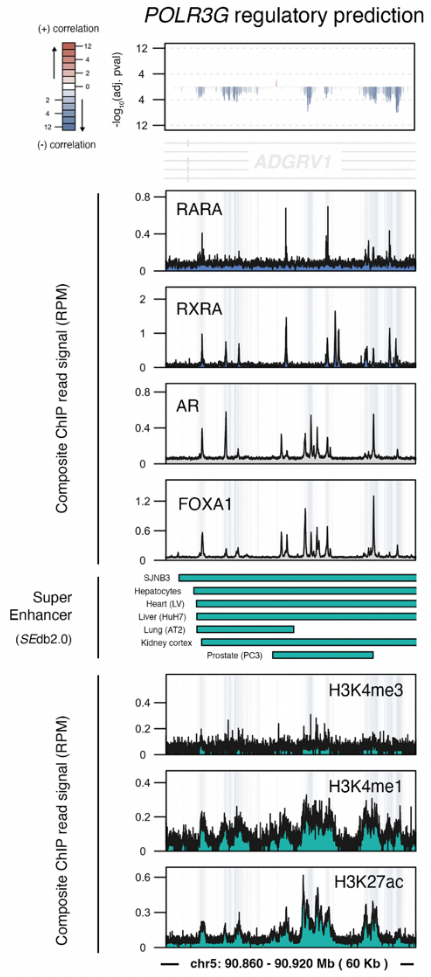

Supplemental Figure S 11. Examination of the strongest negative chromatin correlates with *POLR3G* expression in cancer (top). ChIP-seq profiles for overlapping factors, including RARA, RXRA, Androgen Receptor (AR), FOXA1, histone marks (H3K4me3, H3K4me1, H3K27ac), and annotated super enhancers (SEdb2.0).

Supplemental Figure S 12. *POLR3G* neighboring genes, *CETN3*, *MBLAC2*, *LYSMD3*, and *ADGRV1*, correlate with *POLR3G* expression in cancer. (**a**) Distribution of median z-scores for 4-gene sets with maximum TSS distance equivalent to *CETN3*, *MBLAC2*, *LYSMD3*, and *ADGRV1*. (**b**) Pan-cancer survival comparison of *POLR3G* with neighboring genes, *CETN3*, *MBLAC2*, *LYSMD3*, and *ADGRV1*. (data acquired from tcga-survival.com version 2.0 (Smith & Sheltzer, 2022)).

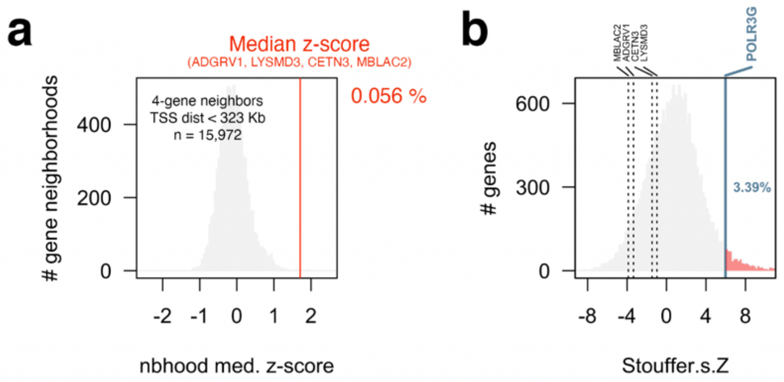

Supplemental Figure S13 caption and figures below.

Supplemental Figure S13. Visualization of transcription factor gene set similarity, as defined by the presence or absence at the promoter (+/- 1 Kb) of 19,022 target genes. Color represents the average ChIP-signal across the top 40 (top 1 bin) genes with highest similarity to *POLR3G*.

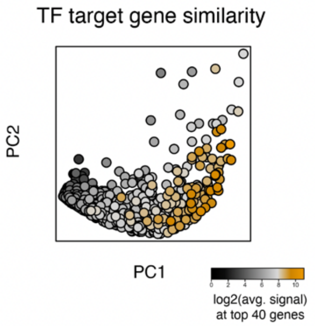

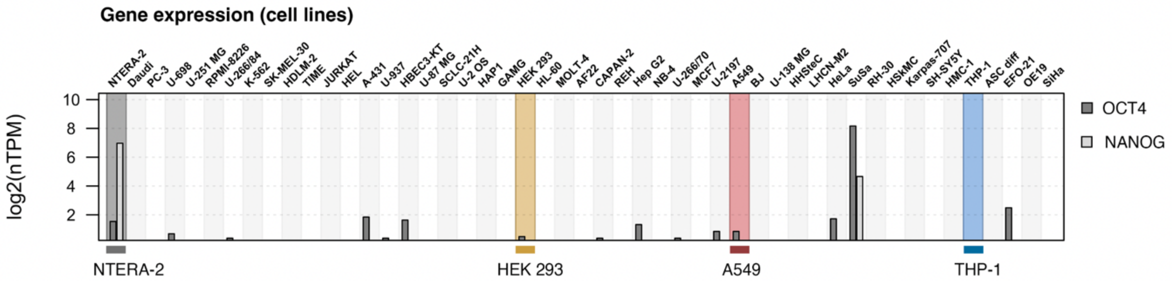

Supplemental Figure S14. Expression of Pluripotency factors OCT4 and NANOG is largely restricted to a limited number of cell lines. OCT4 and NANOG gene expression profiles across ~50 human cell lines (nTPM = normalized transcript per million; proteinatlas.org).

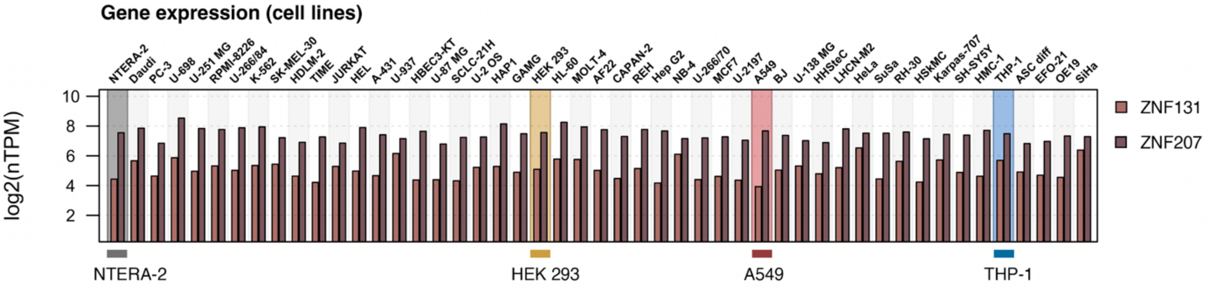

Supplemental Figure S15. Expression of pluripotency-related Zinc finger proteins ZNF131 and ZNF207 is generally ubiquitous across most cell lines. ZNF131 and ZNF207 gene expression profiles across ~50 human cell lines (nTPM = normalized transcript per million; proteinatlas.org).
